# Tele–Cognitive Behavioral Therapy for the Treatment of Diabetes-Related Distress in Individuals With Diabetes Mellitus: Systematic Review and Meta-Analysis of Randomized Controlled Trials

**DOI:** 10.2196/80476

**Published:** 2025-12-24

**Authors:** Xiaohong Xu, Fang Wang, Shunqi Liao, Jingxian Liu, Lingyi Xiao

**Affiliations:** 1 School of Nursing, Chengdu University of Traditional Chinese Medicine, Chengdu, Sichuan, China; 2 Dean's Office, Guang'an Hospital of Traditional Chinese Medicine, 1 Cuiping Road, Guang'an District, Guang'an, Sichuan Province, 638000, China, 86 18980880156; 3 Department of Nursing, Hospital of Chengdu University of Traditional Chinese Medicine, Chengdu, Sichuan, China

**Keywords:** telemedicine, diabetes mellitus, cognitive behavioral therapy, meta-analysis, systematic review

## Abstract

**Background:**

Diabetes-related distress (DD) is highly prevalent in individuals with diabetes mellitus (DM) and impairs quality of life. Tele–cognitive behavioral therapy (Tele-CBT) shows potential for reducing DM-related psychological distress; prior research focused primarily on in-person cognitive behavioral therapy, leaving Tele-CBT’s efficacy poorly characterized.

**Objective:**

This systematic review aims to evaluate Tele-CBT effects on DD, depressive/anxiety symptoms, and hemoglobin A_1c_ (HbA_1c_) levels.

**Methods:**

Eligible studies were randomized controlled trials assessing Tele-CBT for DM-related psychological distress in adults with type 1 or 2 diabetes; in-person cognitive behavioral therapy was excluded. Ten databases (6 English databases and 4 Chinese databases) were searched from inception to May 20, 2025, and updated on September 25, 2025. Two reviewers (XX and SL) independently screened studies, extracted data, and assessed risk of bias using the Cochrane RoB 2.0 tool. In RStudio (Posit Software, PBC), random-effects models incorporating the Hartung-Knapp-Sidik-Jonkman adjustment were used to synthesize effect sizes as standardized mean difference (SMD) with 95% CI. In addition, the quality of evidence was assessed using the GRADE (Grading of Recommendations Assessment, Development, and Evaluation) framework.

**Results:**

A total of 11 randomized controlled trials (n=2467) from 7 countries were included, published between 2017 and 2025. Tele-CBT effectively reduced DD (SMD −0.34, 95% CI −0.58 to −0.09, 95% prediction interval (PI) −0.88 to 0.21), depressive symptoms (SMD −0.66, 95% CI −1.01 to −0.31, 95% PI −1.58 to 0.27), and HbA_1c_ (SMD −0.13, 95% CI −0.25 to −0.01, 95% PI −0.29 to 0.08) compared with controls post intervention, despite the overall evidence being of “low” to “very low” certainty. In addition, no significant effect was observed on anxiety (SMD −0.26, 95% CI −0.71 to 0.19, 95% PI −0.92 to 0.43). Subgroup analysis stratified by intervention duration revealed that interventions lasting >8 weeks were more effective for DD, with a statistically significant difference (*P*<.05) but no significant difference for depressive symptoms (*P*=.31). Metaregression confirmed that neither intervention duration nor the proportion of females was a significant moderator.

**Conclusions:**

This systematic review is the first to quantify the disease-specific efficacy of Tele-CBT for improving DD, depressive symptoms, and HbA_1c_ in people with diabetes, demonstrating its value as an accessible alternative to in-person therapy. By addressing diabetes-specific psychological needs and overcoming practical barriers through remote delivery, Tele-CBT offers a scalable solution for underserved populations. These findings require cautious interpretation due to substantial heterogeneity, moderate risk of bias, and low certainty of evidence. The 95% PIs indicate that real-world benefits may vary considerably by setting or population. Nevertheless, Tele-CBT represents a promising, cost-effective approach to expanding mental health care access. Given its likely variable effectiveness, more evidence is required to determine its value across diverse health care settings.

## Introduction

Diabetes mellitus (DM), a widespread chronic condition, poses a significant public health challenge. The latest findings from *The Lancet* show that from 1990 to 2022,, the global prevalence of DM in adults showed a significant upward trend, rising steadily from 7% to 14% [1]. As of 2022, the number of adults with DM worldwide had increased to approximately 828 million [1]. DM not only directly threatens individuals’ lives through severe complications such as cardiovascular diseases, chronic kidney disease, and ketoacidosis but also exerts a substantial socioeconomic burden. Annual global health care expenditures associated with DM exceed US $966 billion [2].

The biopsychosocial medical model posits that health is shaped by the interplay of biological, psychological, and social factors [3]. DM’s chronic progressive nature subjects individuals to prolonged stress from disease management and prognostic uncertainty, increasing their vulnerability to emotional burdens such as guilt, fear, and worry [4]. Approximately 40% of individuals with DM develop diabetes-related distress (DD) [5]—a condition characterized by emotional exhaustion and behavioral dysregulation in disease management [6]. Epidemiological data show that DM individuals have a 1.33-fold increased risk of depression compared with the general population [7], with a 25% higher prevalence of anxiety [8]. These psychological burdens not only impair individuals’ self-management capabilities in diet, exercise, and medication, affecting quality of life [9], but also induce insulin resistance and accelerate DM progression by triggering hypothalamic-pituitary-adrenal axis dysfunction, autonomic nervous system disorders, and inflammatory responses [10]. Social factors indirectly impact disease progression by influencing psychological states and self-management behaviors. Therefore, early identification and intervention of psychological distress are essential for comprehensive DM management.

Current international diabetes treatment guidelines recommend integrating routine screening for emotional distress, including depression, into standardized DM care and emphasize the importance of appropriate psychological interventions [11]. Recognized as an effective nonpharmacological approach, cognitive behavioral therapy (CBT) has been identified by the World Health Organization as a core intervention in evidence-based medicine for enhancing mental health outcomes in individuals with chronic diseases [12]. CBT, a multicomponent structured psychological intervention, systematically assesses and modifies individuals’ cognitive biases through techniques such as cognitive restructuring, behavioral activation, and problem-solving [13]. By disrupting the negative “cognition-emotion-behavior” cycle, CBT achieves bidirectional regulation of thinking and behavior patterns, ultimately ameliorating psychological outcomes [14]. A meta-analysis [15] encompassing 33 studies demonstrated that standardized CBT yielded a moderate improvement in depressive symptoms among individuals with DM (*d*=0.301, 95% CI 0.115‐0.487; *P*<.001) and a small effect on psychological stress and distress (*d*=0.275, 95% CI 0.068‐0.482; *P*=.014). However, it showed no efficacy for anxiety or hemoglobin A_1c_ (HbA_1c_) levels, likely due to short intervention durations and a predominantly in-person delivery format [15]. Another meta-analysis in individuals with DM and comorbid depression [16] found that CBT effectively alleviated depressive symptoms and reduced fasting blood glucose, while resulting in long-term improvements in quality of life and anxiety. These findings indicate that in-person CBT, as an evidence-based psychological intervention, has positive impacts on DD, depressive and anxiety symptoms, and glycemic control in individuals with DM.

Although in-person CBT has proven effective, its clinical implementation is hindered by several barriers, leading to limited accessibility [17]. Globally, mental health professionals are scarce. The World Health Organization reports [18] that only 45% of the population in high-income countries and 15% in low-income countries have access to psychotherapy, making face-to-face CBT models dependent on professional therapists, challenging to implement in regions with scarce health care resources. Financial constraints further exacerbate the problem. A survey [19] conducted by the Mental Health Commission of Canada in August 2023 revealed that the proportion of individuals unable to afford psychotherapy due to financial constraints increased from 18% to 29% within the past year. In addition, concerns about privacy, stigma, time commitment, and treatment efficacy further deter individuals from seeking psychotherapy [20]. Meanwhile, many individuals report reluctance to receive in-person psychotherapy [21]. Therefore, it is essential to deliver more flexible, accessible, and cost-effective psychotherapy models [16].

Driven by advancements in internet technology and the COVID-19 pandemic, the use of clinical telemedicine increased by 154% year over year from 2019 to late March 2020 [22]. Among them, telepsychotherapy, a key branch of telemedicine, may gradually emerge as a vital alternative to in-person psychotherapy due to its high cost-effectiveness and accessibility advantages [23]. Considering the current limitations of in-person CBT, digital health care technologies have led to the development of tele–cognitive behavioral therapy (Tele-CBT) models. This model incorporates core elements of evidence-based interventions (eg, cognitive restructuring and behavioral activation) while mitigating reliance on professional therapists through technological means. As an evidence-based psychological intervention with proven potential, Tele-CBT provides non–face-to-face psychological support for individuals with DM [20]. It uses internet platforms, mobile apps, telephone communication, and other modalities as delivery channels to translate CBT’s core principles into telehealth-adapted intervention modules. This model supports both minimal-contact and real-time remote guidance from therapists and can be designed as fully self-guided programs [24]. The flexibility of this delivery approach has demonstrated unique advantages in clinical studies of anxiety and depression [25,26]. Compared with traditional in-person CBT models, Tele-CBT alleviates geographic, physical, and temporal limitations. In addition, it reduces labor costs for health care systems through automated modules and improves the potential for cross-regional health care collaboration [27].

A systematic review [17] including multiple disease populations indicated that Tele-CBT and traditional CBT had comparable efficacy for various mental health and somatic disorders. However, Tele-CBT enhances service accessibility through remote delivery, making it particularly suitable for underserved remote or rural regions with scarce health care resources [17]. A meta-analysis by Jenkinson et al [28] in individuals with DM found that Tele-CBT interventions incorporating digital modules significantly outperformed in-person models in improving DD. Although clinical studies have demonstrated the efficacy of Tele-CBT for DD in individuals with DM [20,29], these studies exhibit methodological and design heterogeneity. Moreover, no meta-analysis has been conducted to evaluate the efficacy of Tele-CBT in individuals with DM, leaving the real-world efficacy of Tele-CBT unclear. Thus, this systematic review aims to quantitatively evaluate the clinical outcomes of Tele-CBT compared with control groups (usual care, waitlist control, or placebo) in the improvement of DD, depression, anxiety symptoms, and HbA_1c_ levels in individuals with DM, and to analyze the impact of different intervention durations on efficacy, to provide evidence-based support for clinical guidelines.

## Methods

### Registration

This systematic review strictly adheres to the guidelines of the PRISMA (Preferred Reporting Items for Systematic Reviews and Meta-Analyses) statement.

### Search Strategy

The literature search was initially designed according to our prospectively registered protocol, which restricted inclusion to English language studies and searched 6 English databases. In the final manuscript, we adjusted the search strategy to enhance comprehensiveness and reduce language bias, and these modifications have been clearly reported as post hoc adjustments.

The literature search for this systematic review was reported in accordance with the PRISMA-S statement [30]. A systematic search was conducted using subject terms combined with free terms, covering the following databases: PubMed (NCBI), Embase (Embase.com), Web of Science (Clarivate), Cochrane Library (Wiley), Scopus (Elsevier), PsycINFO (Ovid), WANGFANG (Wanfang Data), CNKI, VIP (VIP Information), and CBM (China Biology Medicine Disc; CBMdisc Platform). The search period was set from the establishment of each database to May 20, 2025. On September 25, 2025, the original search strategies for each database were rerun to update the search. The search strategy of this systematic review was developed by adapting and refining strategies from previous studies [15,28,31], and no dedicated peer review of the search strategy was conducted. Search parameters were set to include only randomized controlled trials (RCTs) and peer-reviewed papers, with no restrictions on publication year or language, and no published search filters were used. To maximize retrieval, citation searching was conducted using the snowball method—specifically, examining both the cited and citing references of all included studies. For missing outcome data in some studies, corresponding authors were contacted via email to supplement the original data not obtained through initial searches. Only the aforementioned methods were used to retrieve literature in this systematic review. No searches of study registries were supplemented, and no purposeful browsing of printed conference proceedings or government websites was conducted. The final search date for all databases is September 25, 2025. The detailed search strategies are shown in Multimedia Appendix 1.

### Inclusion and Exclusion Criteria

Inclusion criteria included the following: (1) Individuals aged 18 years and older with a diagnosis of type 1 diabetes mellitus (T1DM) and type 2 diabetes mellitus (T2DM), excluding those with gestational diabetes or juvenile-onset diabetes. (2) Experimental group interventions were based on Tele-CBT and delivered through remote modalities such as internet, video, telephone, or email modalities. Studies were included if they incorporated CBT’s 2 core components: cognitive restructuring and behavioral activation, including problem-solving skills. Problem-solving therapy—considered a CBT variant and a stand-alone psychotherapeutic approach [32]—was included when used as the psychological intervention. Control groups included waitlist control, usual care, placebo, and others. The minimum intervention period must be at least 4 weeks. (3) Study design: RCTs. (4) Primary outcome measures: DD and depression, with DD assessed using the Problem Areas in Diabetes (PAID) Scale or the Diabetes Distress Scale (DDS); secondary outcomes: anxiety and HbA_1c_. (5) It includes peer-reviewed published journal papers, with no restrictions on language or publication date.

Exclusion criteria included duplicate publications; non–peer-reviewed materials such as study protocols, conference abstracts, and reviews, as these typically lack rigorous methodological validation; studies with inaccessible full texts, unobtainable raw data, or unconvertible data; and studies that either did not measure the target outcomes or measured them but failed to report the results.

### Study Selection and Data Extraction

Two researchers (XX and SL) identified duplicates using EndNote’s duplicate detection strategy and then manually removed duplicate references. They conducted a preliminary review by screening titles and abstracts in strict accordance with predefined inclusion and exclusion criteria, followed by a full-text review. Disagreements were resolved by consultation with a third reviewer (FW). If there are multiple publications for the same study, the latest full report should be preferred. During data extraction, for studies with missing outcome data, we first attempted to contact the authors to obtain the data. If the authors did not respond or the data could not be obtained, such studies were excluded from the meta-analysis. Priority is given to extracting immediate postintervention data, with supplementary extraction of follow-up data for comparative analysis. Double-checking was performed for data extraction of basic information (first author, year), demographic characteristics (country, age, and sex), sample size, basic disease characteristics (type of diabetes, disease course, depressive symptoms, and comorbidities), intervention details (intervention content, therapist, intervention frequency, duration, and follow-up), control groups, outcomes, and intention-to-treat (ITT) analysis.

### Literature Quality Assessment

Two reviewers (XX and JL) independently assessed the methodological quality of the included studies using the 7 domains of the Cochrane Risk of Bias tool (version 2.0). Disagreements were resolved through discussion with a third reviewer (FW). The Cochrane Risk of Bias tool comprises 5 key domains: randomization process, deviation from intended interventions, missing data, outcome assessment, and selective reporting. Each domain is evaluated as “low risk,” “high risk,” or “unclear risk.” Studies were classified as grade A if all domains met “low risk,” grade B if some domains met the criteria, and grade C if none did. We included literature rated only as grade A or B.

### Data Analysis

While the original protocol specified the use of Stata (version 17.0) and model selection based on *I*² and Q test *P* values, the final analysis was conducted in RStudio using a conservative random-effects model grounded in conceptual assumptions about between-study heterogeneity. Effect sizes were pooled using the Hartung-Knapp-Sidik-Jonkman (HKSJ) method. To strengthen methodological rigor and clinical interpretability, additional unprespecified analyses were introduced during the analytical refinement phase, including metaregression, reinterpretation of Egger’s test to assess small study effects, incorporation of 95% prediction interval (PI), quantification of heterogeneity via τ², and GRADE (Grading of Recommendations Assessment, Development, and Evaluation)-based evidence quality assessment.

This systematic review used changes in means and SDs before and after interventions to calculate pooled effect sizes. SD changes were calculated using formulas provided in the Cochrane Handbook. Owing to heterogeneity in outcome measurement tools, standardized mean differences (SMDs) with 95% CIs were used to aggregate effect sizes. Effect sizes were interpreted using Cohen *d* criteria: values of 0.2, 0.5, and 0.8 represent small, medium, and large effects, respectively [33]. Furthermore, we also reported PIs to quantify the real-world effect variability across populations or settings [34]. Notably, traditional PIs often underperform with small study numbers or low heterogeneity [35,36]. Therefore, we used the bootstrap method proposed by Nagashima et al [35] to calculate the PIs. This method draws samples of the heterogeneity parameter τ² from its confidence distribution using the exact distribution of Cochran’s Q statistic. These samples are then combined with the Hartung-Knapp standard errors to build a prediction distribution. This approach provides more accurate coverage even when there are few studies (*K*<5) or moderate heterogeneity, improving the robustness of our PI estimates. This will enhance the robustness of our PI inference. Furthermore, to assess the practical clinical significance of the effect, we sought the minimum important difference reported in previously published studies in this field whenever possible to determine whether our findings hold meaningful value in clinical practice.

The Cochrane Collaboration recommends the HKSJ method for conducting random-effects meta-analyses [37]. When addressing low statistical power linked to having relatively few included studies, the random-effects meta-analysis using the HKSJ method performs better than the standard DerSimonian-Laird method [38]. By using the t-distribution and adjusting the standard error with the q-statistic, the HKSJ method gives more robust CI estimates for effect sizes. Particularly when the number of studies is limited, HKSJ-estimated CIs are more accurate, as they better account for the inherent uncertainty and imprecision in effect estimates [39]. Model selection should be based on conceptual assumptions regarding the distribution of true effects, rather than descriptive statistics such as *I*² [40,41]. Given differences in participant characteristics, intervention content, and participant adherence across the included studies, we used a conservative random-effects model for all analyses. This systematic review uses restricted maximum likelihood estimation to estimate between-study variance, thereby more accurately reflecting the impact of true heterogeneity on effect estimates. Heterogeneity is quantified using Q tests, *I*², and τ² [41]. All statistical analyses were performed using R (version 4.5.0; R Core Team) software in RStudio, and the R packages involved include metafor, forestplot, ggplot2, and so on.

Subgroup analyses of postintervention data were conducted based on a priori intervention criteria: (1) whether professional guidance was provided, and (2) intervention duration. As recommended by Cochrane Handbook guidelines, subgroup analyses were performed when ≥10 studies existed and subgroup distribution was uniform [42]. Metaregression analyses were conducted for primary outcome measures, with intervention duration and proportion of females as moderator variables.

Sensitivity analyses were conducted to evaluate the stability of results. Funnel plots were plotted, and Egger’s regression intercept was used to examine small-study effects. Notably, these methods detect small-study effects rather than directly assessing publication bias [43,44]. The small-study effect may arise from publication bias, selective reporting, genuine heterogeneity among studies, and methodological differences, among other factors. Per methodological standards, reliable interpretation of funnel plots requires 10 or more studies; otherwise, interpretive reliability is low [43,45]. We applied Egger’s regression intercept method to all outcomes. Notably, when the number of studies is small, statistical power is significantly reduced, which may lead to false-negative results [44]. The final published outcome measures in this review are fully consistent with the prespecified outcome measures.

### Evidence Grade Evaluation

Two researchers (XX and SL) independently evaluated the overall quality of the included outcome indicators using GRADEpro software (Evidence Prime Inc, in affiliation with McMaster University and the GRADE Working Group). The GRADE tool classifies evidence quality into 4 levels: high, moderate, low, and very low. Factors leading to downgrading include 5 aspects: risk of bias, imprecision, inconsistency, indirectness, and publication bias [46]. High-quality evidence indicates very high certainty that the true effect is close to the estimated effect. Moderate-quality evidence reflects moderate confidence in the effect estimate, where the true value is likely close to the estimate but may differ substantially. Low-quality evidence indicates limited certainty in the effect estimate, and the true value may differ markedly from the estimate. Very low-quality evidence suggests almost no confidence in the effect estimate, and the true value is very likely to differ substantially from the estimate.

## Results

### Literature Search Results

**Figure 1.**
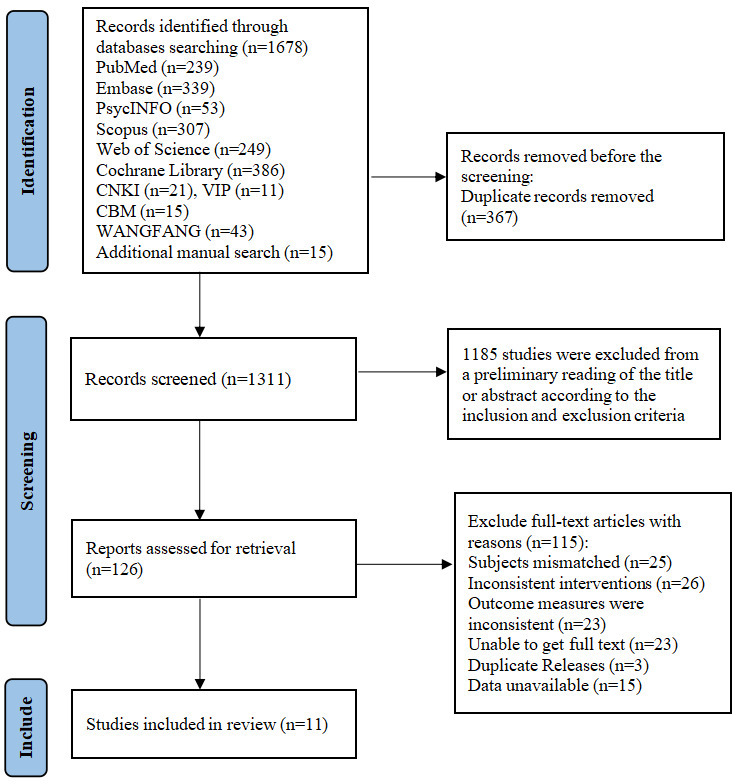
Flowchart of literature screening. CBM: China Biology Medicine; CNKI: China National Knowledge Infrastructure; VIP: VIP Information.

An initial systematic database search yielded 1678 studies. After removing 367 duplicate studies, 1311 studies remained. Following title and abstract screening, 126 studies underwent full-text review. A total of 115 studies were excluded after full-text evaluation, including 25 with inappropriate study populations, 26 with inconsistent interventions, 23 with different outcome measures, 23 without accessible full text, 3 duplicate publications, and 15 with unobtainable or unconvertible data. Ultimately, 11 studies were included [20,29,47-55]. The flowchart of literature screening is shown in Figure 1.

### Characteristics of Included Studies

A total of 11 RCTs involving 2467 individuals with DM were included (Multimedia Appendix 2). These studies were published between 2017 and 2025. The study populations originated from South Korea (n=1) [47], Australia (n=3) [20,29,50], the Netherlands (n=1) [49], Spain (n=1) [48], Germany (n=2) [51,53], the United States (n=2) [52,55], and the United Kingdom (n=1) [54]. The mean age of the participants ranged from 34 to 80 years, and all studies were 2-arm trials. Among them, 4 studies primarily focused on people with T2DM [20,47,49,52], 2 studies included people with T1DM [48,54], and 5 studies did not clearly differentiate the types of diabetes [29,50,51,53,55]. The studies included people with varying degrees of depressive symptoms, and some reported information on related complications. Tele-CBT interventions were delivered via online platforms, mobile apps, telephone, or email. Control groups included waitlist control, usual care, or placebo. A total of 10 studies [29,47-55] provided professional guidance conducted by trained nurses, PhD-level researchers, or psychologists; only 1 study [20] was fully self-guided. In terms of intervention implementation, Tele-CBT followed a structured design with varied frequency and duration across studies. Most adopted a weekly frequency, with single-session duration ranging from 15 to 60 minutes. Intervention duration ranged from 6 weeks to 12 months, with most focusing on 8‐ to 12-week medium-term programs. While 6 studies reported follow-up, only 3 [49,53,55] provided complete outcome data for quantitative synthesis. In statistical analysis, 10 of the 11 studies implemented ITT analyses to minimize selection bias.

### Methodological Quality Assessment

**Figure 2.**
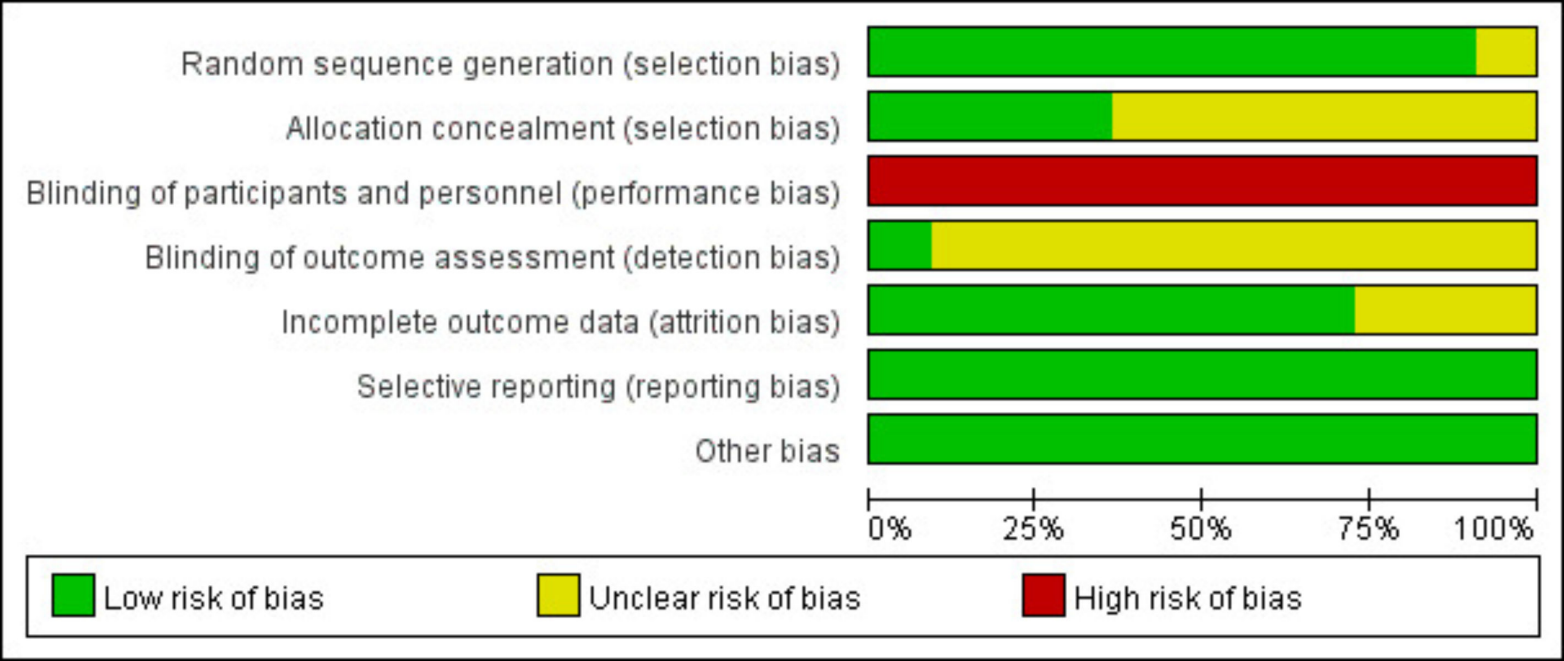
Risk of bias graph.

A total of 10 studies reported randomization methods for allocation, and 4 studies described allocation concealment methods. Because Tele-CBT is a type of psychological intervention, blinding participants and practitioners was difficult; as a result, none of the studies used blinding, which raises a high risk of implementation bias. Only 1 study mentioned blinding outcome assessors, while the remaining studies did not describe this process well enough. Also, since outcome measures were mainly assessed using subjective scales, the risk of measurement bias is still uncertain. For handling incomplete outcome data, 10 studies used ITT analysis. No studies showed selective reporting bias or other types of bias. All included studies were rated as grade B (Figures2  3).

**Figure 3.**
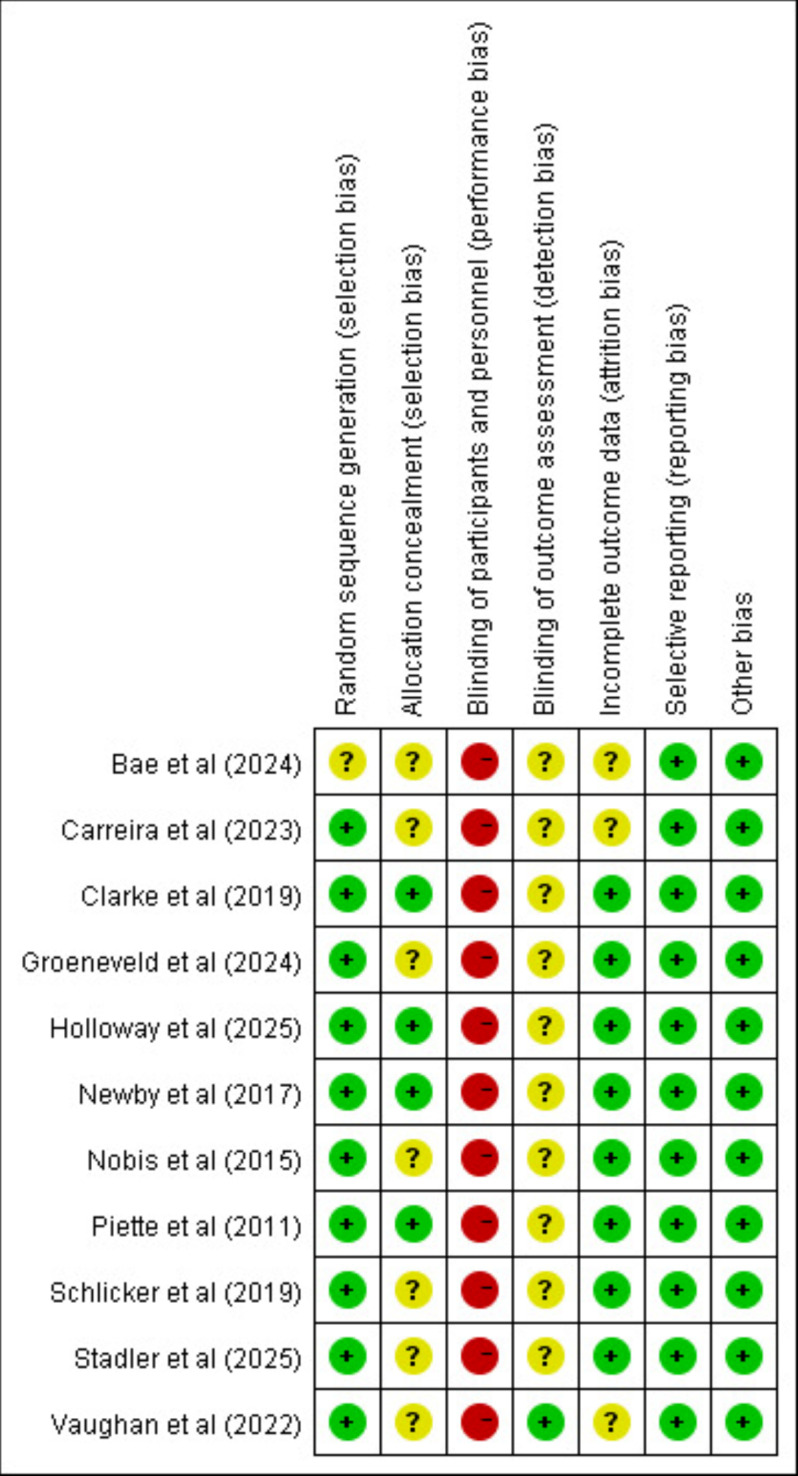
Risk of bias summary [20,29,47-55]

### Meta-Analysis Results

#### Diabetes-Related Distress

**Figure 4.**
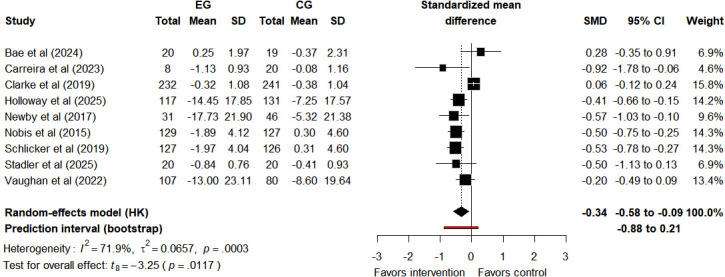
Forest plot of diabetes-related distress results assessed after the intervention [20,29,47,48,50,51,53-55]. CG: control group; EG: experimental group; HK: Hartung-Knapp (method); SMD: standardized mean difference.

Nine studies [20,29,47,48,50,51,53-55] reported data on DD, with 2 [53,55] providing follow-up information. After the Tele-CBT intervention, there was a small degree of improvement in DD (SMD −0.34, 95% CI −0.58 to −0.09, 95% PI −0.88 to 0.21; *I*²=71.9%, τ^2^=0.0657, *P*<.001) (Figure 4). The 95% CI confirms the statistical significance of the average effect. However, the 95% PI includes zero, indicating potential variability. Tele-CBT may have no effect on DD or even show nonbeneficial trends in some diabetic populations. During long-term follow-up, the average effect diminished (SMD −0.20, 95% CI −1.53 to 1.13, 95% PI −1.43 to 1.14; *I*²=9.7%, τ^2^=0.0024, *P*=.29) (Figure S1 in Multimedia Appendix 3). Both the 95% CI and 95% PI include zero and have wide ranges: the former indicates a lack of statistical reliability for the average effect, while the latter reflects variability in future effects, collectively suggesting uncertainty in both types of effects. Note that due to the limited number of studies, the 95% PI may be unreliable. For the pooled postintervention effect size, using the leave-one-out exclusion method, after excluding the study by Clarke et al [20], the results of the meta-analysis changed significantly (SMD −0.42, 95% CI −0.61 to −0.22, 95% PI −0.77 to −0.05; *I*²=28.6%, τ²=0.0141, *P*=.18) (Figure S2 in Multimedia Appendix 3). The quality of evidence for the effect of Tele-CBT on DD after the intervention was rated as “very low” (Multimedia Appendix 4). Downgrading was performed due to increased risks of methodological bias, inconsistency, and imprecision. In postintervention assessments, Tele-CBT had minimal impact on DD.

To address the high heterogeneity in immediate postintervention DD improvement, we conducted an observational subgroup analysis stratified by intervention duration to identify potential moderating factors. Subgroup results showed that the DD improvement effect was greater in the >8 weeks subgroup (SMD −0.51, 95% CI −0.67 to −0.36, 95% PI −0.70 to −0.31; *P*=.006) than in the ≤8 weeks subgroup (SMD −0.27, 95% CI −0.59 to 0.06, 95% PI −0.83 to 0.31; *P*=.90; Figure S3 in Multimedia Appendix 3); the subgroup difference test confirmed a statistically significant difference between the 2 subgroups (*χ*²_1_=3.86; *P*<.05). Notably, this subgroup analysis is a post hoc stratification that lacks original RCT randomization, so confounding factors cannot be rigorously controlled. The observed DD improvement difference is thus observational and not causal evidence that intervention duration drives such differences. Since the self-guided group included only 1 study, subgroup analysis for this dimension was not conducted according to the prior design. Metaregression results suggested that intervention duration (β=.013, SE=0.017, 95% CI −0.03 to 0.05; *P*=.49; Figure S4 in Multimedia Appendix 3) and proportion of females (β=−.004, SE=0.005, 95% CI −0.02 to 0.01; *P*=.40; Figure S5 in Multimedia Appendix 3) might not be moderators affecting intervention effects. Due to the limitation of the number of studies, statistical power might be insufficient, and these subgroup and metaregression findings should be interpreted with caution.

#### Depressive Symptoms

**Figure 5.**
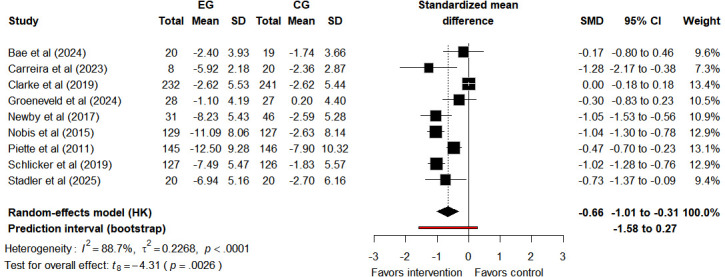
Forest plot of depression outcomes after intervention assessment [20,29,47-49,51-54]. CG: control group; EG: experimental group; HK: Hartung-Knapp (method); SMD: standardized mean difference.

Nine studies [20,29,47-49,51-54] reported depressive symptoms, with 2 studies [49,53] providing follow-up data. Meta-analysis showed a moderate average effect of Tele-CBT on reducing depressive symptoms post intervention (SMD −0.66, 95% CI −1.01 to −0.31, 95% PI −1.58 to 0.27; *I*²=88.7%, τ²=0.2268, *P*<.001; Figure 5). The 95% CI confirms its statistically significant average effect, but the 95% PI crosses zero—Tele-CBT may have no effect or be harmful in some populations. Note that the number of included studies is small, which could compromise the accuracy of estimating future effect variability; this 95% PI may be unreliable. The results from the leave-one-out sensitivity analysis show that when the study by Clarke et al [20] is excluded, the pooled effect estimate changed (SMD −0.76, 95% CI −1.08 to −0.45, 95% PI −1.42 to −0.12; *I*²=69.8%, τ²=0.0884, *P*=.001; Figure S6 in Multimedia Appendix 3). Such a change indicates that the stability of the original meta-analysis results is affected by this study, and the overall stability requires careful consideration. During follow-up, the effect persisted numerically (SMD −0.63, 95% CI −1.59 to 0.34, 95% PI −1.57 to 0.45; *I*²=0%, τ²=0, *P*=.51; Figure S7 in Multimedia Appendix 3). The 95% CI crosses zero, so the average effect is not statistically significant. Consistently, the 95% PI also crosses zero, reflecting high uncertainty in future effects. Similarly, the reliability of this 95% PI should be cautious, especially with few follow-up studies. The quality of evidence for Tele-CBT on depression is rated as “low” due to methodological bias risk and imprecision (Multimedia Appendix 4). In postintervention assessments, the impact of Tele-CBT on depression is small.

To explore the potential impact of intervention duration on the improvement of depressive symptoms, we conducted an exploratory subgroup analysis stratified by intervention duration. Subgroup results showed that the improvement effect of depressive symptoms was numerically greater in the >8 weeks subgroup (SMD −0.87, 95% CI −1.80 to 0.06, 95% PI −1.92 to 0.08; *P*<.001), while the ≤8 weeks subgroup showed a statistically significant improvement in depressive symptoms (SMD −0.55, 95% CI −1.06 to −0.04, 95% PI −1.38 to 0.33; *P*=.04; Figure S8 in Multimedia Appendix 3). However, the subgroup difference test found no statistically significant difference in improvement effects between the 2 subgroups (*χ*²_1_=1.05; *P*=.31). This subgroup analysis is a post hoc stratification. This nonsignificant difference is merely an exploratory observational pattern and not definitive evidence that intervention duration has no effect on depressive symptom improvement. There was only 1 study on self-directed groups, so subgroup analysis was not conducted according to the prespecified guidance types. Metaregression results indicated that intervention duration *(*β=.007, SE=0.012, 95% CI −0.02 to 0.03; *P*=.54; Figure S9 in Multimedia Appendix 3) and proportion of females (β=−.011, SE=0.010, 95% CI −0.03 to 0.01; *P*=.29; Figure S10 in Multimedia Appendix 3) might not affect intervention effects. Due to the limited number of included studies, the statistical power of metaregression was reduced, and the research results should be interpreted with caution.

#### Anxiety

**Figure 6.**
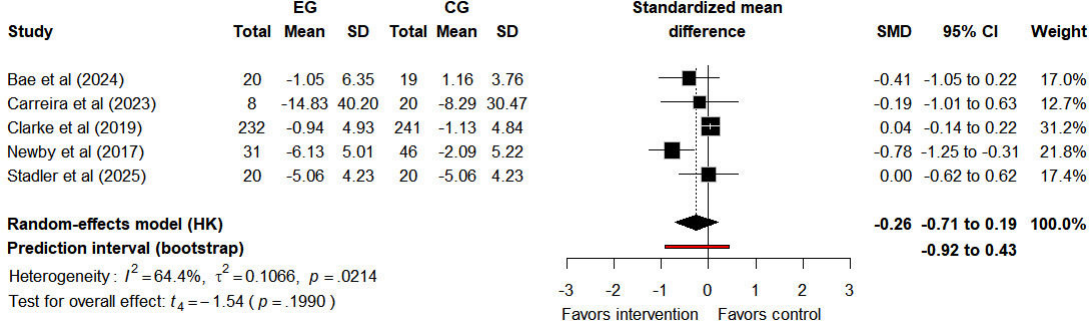
Forest plot of anxiety outcomes after intervention assessment [20,29,47,48,54]. CG: control group; EG: experimental group; HK: Hartung-Knapp (method); SMD: standardized mean difference.

Anxiety symptoms were evaluated in 5 studies [20,29,47,48,54]. Results indicated minimal to no effect of Tele-CBT on anxiety (SMD −0.26, 95% CI −0.71 to 0.19, 95% PI −0.92 to 0.43; *I*²=64.4%, τ^2^=0.1066, *P*=.02; Figure 6). The 95% CI crosses zero, confirming that the average effect of Tele-CBT on anxiety lacks statistical significance. The 95% PI is wide and also crosses zero, reflecting high variability in potential effects. In specific populations or future studies, Tele-CBT may still show no obvious effect on anxiety or even nonbeneficial trends. Note that the number of included studies is small, so this 95% PI may be unreliable, as limited data could reduce the accuracy of estimating future effect distribution. Combined with *I*² and τ², this substantiates notable absolute heterogeneity in true effects across studies, unrelated to random sampling error. After excluding the study by Clarke et al [20] in the sensitivity analysis, the intervention effect of Tele-CBT on anxiety showed no substantial change (SMD −0.41, 95% CI −0.98 to 0.17, 95% PI −1.27 to 0.46; *I*²=29.7%, τ²=0.0446, *P*=.23; Figure S11 in Multimedia Appendix 3), which further indicates that the original meta-analysis results exhibit good stability. Since there was no significant effect of the intervention on anxiety indicators and the number of studies was limited, we did not perform a GRADE assessment for it.

#### Hemoglobin A_1c_

**Figure 7.**
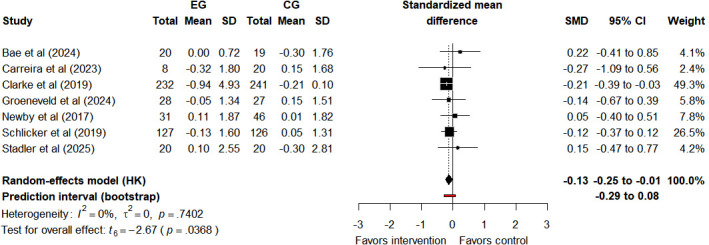
Forest plot of hemoglobin A_1c _outcomes after intervention assessment [20,29,47-49,53,54]. CG: control group; EG: experimental group; HK: Hartung-Knapp (method); SMD: standardized mean difference.

Seven studies [20,29,47-49,53,54] measured HbA_1c_, with 1 study [49] providing follow-up data. Although the heterogeneity statistic *I*²=0%, we used a random-effects model to provide more conservative and generalizable effect estimates. This decision is based on the differences in participant characteristics and intervention content across the included studies. Compared with control groups, Tele-CBT interventions resulted in minimal improvements in HbA_1c_ (SMD −0.13, 95% CI −0.25 to −0.01, 95% PI −0.29 to 0.08; *I*²=0%, τ²=0, *P*=.74; Figure 7). The 95% CI does not cross zero, confirming the statistically significant minimal average improvement of Tele-CBT on HbA_1c_, with no heterogeneity among studies (*I*²=0%). However, the 95% PI crosses zero, indicating that in future studies or different populations, Tele-CBT may have no improvement effect on HbA_1c_. Note that the number of included studies is small, this 95% PI may be unreliable. The quality of evidence for HbA_1c_ after the intervention is “very low” (Multimedia Appendix 4). The rating was downgraded due to risk of bias, inconsistency, and imprecision. Tele-CBT has little effect on HbA_1c_.

### Small Study Effects Assessment

To assess small study effects, we combined visual inspection of funnel plots and Egger’s regression intercept test in accordance with the methodological guidance from Sterne et al [56]. The number of included studies for all outcomes of interest was <10, which is below the ideal sample size threshold (≥10 studies) for reliable small-study effect assessment as noted by Sterne et al [56]. Nevertheless, funnel plots (Figures S12-S15 in Multimedia Appendix 3) were constructed to visually explore potential asymmetry. Visual inspection revealed no obvious asymmetry across outcomes, although the limited number of studies introduces greater uncertainty into this visual judgment. Egger’s regression intercept test was further used to quantify small-study effects, and no significant small-study effects were detected for any outcome: DD (*z*=−0.685; *P*=.50), depression (*z*=−0.629; *P*=.53), anxiety (*z*=−0.503; *P*=.62), and HbA_1c_ (*z*=1.245; *P*=.21). These results must be interpreted with caution. Sterne et al [56] pointed out that fewer than 10 studies reduce the statistical power of Egger’s test, increasing the risk of failing to detect true small-study effects. Lau et al [43] further noted that funnel plot symmetry or a nonsignificant Egger’s test does not rule out publication bias. In addition, if funnel plot asymmetry were present, it could stem from heterogeneity or methodological flaws rather than publication bias alone [56]. Therefore, the current results cannot exclude publication bias or selective reporting bias, which may remain undetected due to the small number of included studies.

## Discussion

### Principal Findings

This systematic review presents the first systematic review and meta-analysis evaluating the efficacy of Tele-CBT in individuals with DM. 11 studies were included, all with moderate bias risk, and overall quality was acceptable. Based on current evidence, this systematic review offers robust evidence for Tele-CBT as an effective psychological intervention for individuals with DM. Tele-CBT produced significant short-term improvements in DD and depressive symptoms, with no statistically significant effects observed at follow-up. For HbA_1c_ levels, Tele-CBT produced a modest yet statistically significant reduction, while no statistically significant effects were found for anxiety symptoms. Subgroup analyses revealed that intervention effectiveness may be influenced by duration. Heterogeneity in intervention protocols and assessment tools across included studies may have impacted the reliability of the results.

Specifically, individuals with DM who received Tele-CBT interventions exhibited small to moderate effect sizes for DD and depressive symptoms, and this aligns with prior studies [47,48]. From the perspective of specific effects and real-world effect variability, the 95% PI for DD is −0.88 to 0.21, which includes 0. This indicates that although the average effects from current research show that Tele-CBT has a positive impact on DD, in future real-world applications, the treatment outcomes may be considerably uncertain due to factors such as differences in population characteristics, variations in clinical settings, and changes in implementation conditions. At the clinical significance level, this effect size for DD (SMD=−0.34) exceeded the minimal clinically important difference (0.25) set for the DDS-17 [57], indicating clinically meaningful improvement. Combined with the characteristic that the PI consistently points to an improvement effect, this suggests that the clinical benefits of Tele-CBT for DD may be universal. However, because the PAID Scale was also used in this systematic review, this suggests that there may be actual differences in its clinical benefits. For depressive symptoms, the 95% PI after Tele-CBT intervention is −1.58 to 0.27, which includes zero. This suggests that although the average effect indicates an improvement in depressive symptoms, the actual effect in new studies or practical applications could range from significant improvement to negligible, or even slightly adverse outcomes. The wide range and inclusion of zero in the PI reflect substantial heterogeneity among studies and indicate that various factors may influence the effectiveness of the intervention. However, no direct minimum important difference was identified for depression, anxiety, and HbA_1c_, so the clinical significance of the improvement effects for these outcomes cannot be directly determined. Notably, both DD and depressive symptoms exhibited varying degrees of interstudy heterogeneity. This suggests that when applying Tele-CBT in clinical practice, full consideration should be given to individual characteristics (eg, age, disease duration, and adherence) and details of the intervention protocol (eg, guidance mode and treatment duration) to maximize the intervention effect.

In this systematic review, depressive symptoms exhibited high heterogeneity (*I*²=88.7%), which may be linked to the intervention protocols. Although all studies used Tele-CBT, there were notable differences in guidance models and content. The study by Clarke et al [20] was the only one using a fully automated self-guided intervention program, with lower treatment intensity than other studies that included professional guidance [29]. This self-guided design lacked diabetes-specific intervention modules, potentially leading to insufficient targeting of emotional distress among people with DM. Meanwhile, the final participant retention rate of 58.4% may have increased heterogeneity and introduced attrition bias [20]. This low retention rate may be linked to the tendency of self-directed interventions to trigger symptom exacerbation, which increases the risk of loss [58]. Additional sources of heterogeneity may include differences between measurement tools (eg, PHQ-9 and BDI) and variations in adherence and comorbidities associated with the broad age range of participants. In sensitivity analysis, excluding the study by Clarke et al [20] reduced the *I*² for depressive symptom outcomes to 69.8% and increased the pooled effect size (SMD −0.76, 95% CI −1.08 to −0.45, 95% PI −1.42 to −0.12). This result not only confirms the robustness of professional-led Tele-CBT in improving depressive symptoms among people with DM but also indicates that guidance type may be one of the sources of heterogeneity.

Looking further, therapist-guided Tele-CBT may offer additional benefits compared with self-directed programs. Professional therapists offer guidance based on individual feedback, dynamically tailoring intervention protocols and delivering tailored intervention modules for people with different thinking patterns and cognitive habits—all to maximize intervention efficacy as much as possible. Moreover, support from professional therapists enhances treatment motivation and adherence [59], thereby improving the efficacy of Tele-CBT interventions. In contrast, self-guided Tele-CBT protocols improve accessibility and optimize cost-effectiveness, although clinical efficacy does not surpass that of professionally guided groups, a finding confirmed in prior studies [60]. Hwang et al [61] also showed that therapist-delivered Tele-CBT is superior to self-guided Tele-CBT. Given that only 1 study was included in the self-guided group in this study, we did not conduct a formal subgroup analysis to compare the effectiveness of different guidance modalities. In future research, it will be necessary to evaluate whether more efficient professional guidance enhances intervention efficacy.

Beyond guidance type, intervention duration, and participant sex may also contribute to heterogeneity in DD and depressive symptoms. We therefore conducted subgroup analyses and metaregression to further explore these potential moderators. Subgroup analyses observed potential differential associations between intervention duration and outcomes. For DD, >8 weeks of interventions showed a more distinct positive improvement effect. The 95% PI (−0.70 to −0.31) for this subgroup further confirms that even when extended to different populations or clinical settings in the future, the improvement effect of long-term Tele-CBT will still likely lean toward a positive direction. However, the span of the interval indicates significant variability in the magnitude of the short-term intervention effect, and such variability requires further evaluation based on patient-specific characteristics (eg, disease duration and presence of comorbidities). For depressive symptoms, ≤8 weeks demonstrated statistically significant improvement. Even so, the 95% PI (−1.38 to 0.33) reflected high variability in short-term effects, suggesting that the improvement effect of short-duration Tele-CBT may vary greatly across different populations or settings. This variability may similarly be linked to small subgroup sizes and unmeasured factors, which may further exacerbate effect heterogeneity. Notably, nonsignificant differences between short-term and long-term effects for depression do not confirm the absence of a duration effect; they merely highlight that post hoc stratification may have limited ability to detect true subgroup differences. All subgroup analyses are inherently observational. They deviate from the original randomization framework and suffer from small sample sizes. These limitations narrow the generalizability of subgroup conclusions, as observed differences should not guide clinical decisions about Tele-CBT duration for diabetes-related outcomes. Instead, their core value lies in generating hypotheses. Future dedicated RCTs that take intervention duration as the primary variable are needed to validate these exploratory findings.

We further performed metaregression analyses to explore whether intervention duration and proportion of females moderated Tele-CBT efficacy on DD and depressive symptoms. For DD, metaregression showed no significant associations between intervention duration or female proportion and effect size. Similar nonsignificant findings were observed for depressive symptoms. However, these results warrant extreme caution, primarily due to low statistical power from the small number of included studies. Methodological guidelines recommend at least 10 studies for metaregression to detect moderate moderator effects with acceptable power; fewer than 10 studies substantially increase the risk of type II errors [62]. In our analysis, the small sample size resulted in wide standard errors and 95% CI spanning zero—we cannot rule out that intervention duration or female proportion exerts true moderating effects, only that our data lacked power to identify them. For instance, the β coefficient for DD intervention duration (β=.013) suggests a weak positive association, but the wide CI and high *P* value reflect uncertainty, not a definitive absence of effect. In addition, metaregression relies on unverifiable assumptions, which are particularly difficult to validate with few studies [62,63]. Unmeasured factors across our included RCTs may also have confounded results, further reducing reliability. Notably, metaregression findings are hypothesis-generating and not causal proof [64]; current results do not confirm these variables as nonmoderators. Future studies should increase the number of high-quality RCTs and prespecify moderators in protocols to robustly test their influence on Tele-CBT efficacy.

Although Tele-CBT shows sustained benefits for DD and depressive symptoms, its effect on anxiety, another common psychological outcome in diabetes, was not significant, contradicting previous findings [29,54]. Notably, sensitivity analysis excluding the study by Clarke et al [20] revealed a substantial shift in effect size, suggesting potential unreliability of the initial conclusions. For anxiety symptoms, the 95% PI is −1.27 to 0.46. In real-world interventions, the effectiveness of Tele-CBT may vary substantially across factors such as intervention protocols and population characteristics and may even be ineffective in some scenarios. Seven studies included HbA_1c_ as an outcome measure. Pooled effect sizes showed a decrease in HbA_1c_ levels following Tele-CBT interventions compared with control groups, contradicting results from previous meta-analyses of in-person CBT [15,28]. This suggests that differences in intervention delivery formats may lead to divergent outcomes. Tele-CBT, which relies on individual self-management, enhances self-monitoring and awareness, facilitating HbA_1c_ control. In this meta-analysis, the 95% CI was −0.25 to −0.01, indicating a consistent average benefit. However, the 95% PI was −0.29 to 0.08, including zero, suggesting that effects may vary across settings and some populations might experience little or no improvement. While the overall evidence supports efficacy, the wider PI reflects uncertainty due to heterogeneity. Given the limited number of studies, more RCTs are needed to confirm under which conditions Tele-CBT reliably improves HbA_1c_.

This systematic review validated that Tele-CBT interventions improve both psychological outcomes and glycemic control in individuals with DM, a finding consistent with the bidirectional pathophysiology linking psychological distress and metabolic dysfunction. A meta-analysis [65] reported a significant association between depressive symptoms, DD, and glycemic control (*r*=0.23, 95% CI 0.15-0.31; *P*<.001). The underlying mechanism involves how depressive symptoms, DD, and anxiety undermine self-management behaviors (eg, medication adherence and glycemic monitoring) [66], whereby negative thinking induces behavioral inertia, exacerbating glycemic fluctuations. Khawagi et al [10] demonstrated that insulin signaling dysregulation in T2DM impairs brain serotonin signaling, increasing depression risk; conversely, depressive states disrupt insulin homeostasis via neuroendocrine mechanisms, establishing a bidirectional relationship between metabolic and psychological pathways. Notably, DD impacts HbA_1c_ levels through self-management behaviors. Tele-CBT alters psychological states by correcting cognitive distortions and indirectly improves metabolic parameters through enhanced self-management [28]. These multidimensional improvements in psychological, behavioral, and metabolic outcomes may be more substantial in subgroups with higher baseline DD scores; future studies should test this hypothesis. Guided by the biopsychosocial model, Tele-CBT enhances patient-provider interactions using technology, enabling comprehensive regulation of treatment burden, emotional distress, and metabolic dysfunction. This approach combines accessibility and efficacy for psychological interventions in diabetes care.

Based on cognitive behavior theory [67], CBT improves the negative cognitive-emotional-behavioral cycle in people with DM through 3 core techniques: cognitive restructuring, behavioral activation, and problem-solving. Different studies vary in their focus on disease-specific characteristics and management needs of people with DM, leading to differences in clinical efficacy. Among these, diabetes-specific modules are the most targeted. Nobis et al [51] and Schlicker et al [53] integrate CBT techniques with diabetes-specific scenarios, focusing on behavioral activation and problem-solving. Key content includes diabetes-specific themes such as concerns about complications, blood glucose monitoring, and doctor-patient communication. Stadler et al [54] further addressed differences in DM subtypes by integrating T1DM-specific modules (eg, hypoglycemia fear, insulin adjustment, and binge eating) into conventional CBT. This approach allows CBT to directly address people’s daily disease management challenges, reflecting that DD comes from the pressures of managing the disease. In contrast, generalized modules focus on addressing basic emotional needs through core CBT techniques. Clarke et al [20] developed automated interaction modules incorporating cognitive and behavioral self-monitoring functions. While these can improve fundamental emotional issues such as depression and anxiety, their intervention effectiveness for DD may be somewhat limited due to the absence of diabetes-specific content. In addition, targeted problem-focused programs provide CBT support specifically addressing particular concerns of people with DM. Groeneveld et al [49] integrated mental health education, sleep restriction, relaxation training, and other techniques to improve secondary emotional issues associated with insomnia and emotional comorbidity. Another example is the low-intensity, professional-led problem-solving therapy mentioned in studies such as Holloway et al [50], which guides people to address specific issues causing distress in disease management. Research that integrates specific scenarios with problem-focused approaches yields significant positive effects.

The CBT intervention content in these studies aligns with the diabetes-specific distress framework under the Biopsychosocial Model [68], which emphasizes that stressors such as treatment burden, fear of complications, and negative patient-provider interactions trigger DD. Tele-CBT intervenes directly on these core stressors by reducing treatment encounter stress through non–face-to-face guidance while increasing patient-provider interaction frequency via technological tools. In regions with high health care expectations, this model may mitigate the exacerbation of DD caused by unmet care needs. Notably, recent studies confirm that collaborative communication significantly improves DD outcomes [68], a finding that further supports why Tele-CBT’s approach to refining patient-provider interactions is effective. Ultimately, this communication-focused model makes psychological support more accessible.

### Limitations

This systematic review has several limitations. First, only 11 studies were included, and the small number of studies may result in imprecise 95% PI and estimation bias, undermining the reliability of the results. In addition, low statistical power due to the limited number of studies may have missed potential small study effects. While current results provide insufficient evidence for small study effects, we cannot entirely rule out the impact of publication bias or selective reporting bias.

Second, this meta-analysis did not strictly differentiate between T1DM and T2DM, which may hide differences in the efficacy of Tele-CBT for each type of diabetes. T1DM is an autoimmune disorder characterized by an absolute deficiency in endogenous insulin secretion, and it usually develops during adolescence. In contrast, T2DM is associated with insulin resistance and is often related to obesity and lifestyle factors [69]. These differences may account for the distinct triggers of DD. At the sociopsychological level, people with T1DM are more likely to have identity concerns that come from the public nature of their disease management behaviors, while those with T2DM often experience self-stigma caused by the social stigma related to lifestyle diseases [70]. This analysis, which does not stratify by diabetes type, may cause Tele-CBT to fail to align well with the specific psychological stressors of the 2 patient groups, thereby potentially affecting the intervention’s efficacy. This finding calls for cautious interpretation of the findings, and future studies should conduct separate subgroup analyses for T1DM and T2DM.

In addition, in this systematic review, outcome assessments relied on self-report scales. Although we used established scales to reduce error, subjective bias remains inherent. Moreover, because psychological interventions involve subjective evaluations, it was hard to use blinding in the study. This increases the risk of detection bias and may affect how objectively we interpret the results. Finally, this systematic review confirms the short-term efficacy of Tele-CBT for people with DM, laying the groundwork for future exploration of its long-term effects. Current studies are limited by inconsistent intervention protocols (content, frequency, and duration) and short follow-up periods that limit the assessment of long-term efficacy. Future research should focus on 3 areas: standardizing intervention protocols through optimizing key parameters, including core modules, guidance frequency, and intervention duration; extending follow-up periods to assess treatment sustainability; and expanding sample sizes and conducting more high-quality RCTs to provide reliable evidence for the long-term clinical application of Tele-CBT.

### Conclusions

This systematic review is the first to synthesize evidence from studies focused on Tele-CBT’s disease-specific value for diabetes care. It confirms that Tele-CBT, on average, outperforms control groups by improving DD and depressive symptoms in people with DM post intervention and also reducing HbA_1c_ levels. However, this conclusion requires nuanced interpretation: the wide PI, which includes values indicating no significant benefit, suggests substantial variability in efficacy across studies, with potential for nonsignificant effects in specific populations or settings. This systematic review innovatively applies Tele-CBT to address diabetes-specific psychological needs of people with diabetes, rather than generalized psychological distress. Second, it highlights how Tele-CBT addresses the practical barriers of in-person CBT through multimodal delivery and professional guidance, thereby enhancing accessibility—particularly in regions with scarce mental health resources. Furthermore, it links Tele-CBT to metabolic control, confirms its dual benefits for mental well-being and glycemic management, and extends Tele-CBT from a stand-alone psychological intervention to a component of comprehensive diabetes care.

Notably, findings require rigorous interpretation alongside methodological context. High heterogeneity for DD and depressive symptoms, combined with the wide PI, limits the broad generalization of the conclusion. Most RCTs had moderate bias, including unfeasible participant and practitioner blinding and unclear allocation concealment—factors that may overestimate Tele-CBT’s actual efficacy. Per GRADE assessment, evidence for DD, depressive symptoms, and HbA_1c_ was rated very low to low, reflecting risks of bias, inconsistency, and imprecision, which collectively weaken confidence in the observed benefits. These limitations urge caution in asserting Tele-CBT’s superiority over control groups.

Despite the study’s limitations, Tele-CBT emerges as a promising strategy to expand accessible and cost-effective mental health support in diabetes care. It could help bridge treatment gaps for individuals in underserved areas or those constrained by time, geography, or privacy concerns. Future research should prioritize standardized Tele-CBT protocols, high-quality RCTs, and longer follow-up to validate efficacy. Such efforts will strengthen evidence for Tele-CBT’s integration into routine diabetes management. While it may not replace all in-person psychological care, Tele-CBT is a promising tool to the unmet mental health needs of people with DM globally.

## Supplementary material

10.2196/80476 Multimedia Appendix 1 Search strategy.

10.2196/80476 Multimedia Appendix 2 Basic characteristics of included literature.[Aff aff1]

10.2196/80476 Multimedia Appendix 3 Subgroup analysis, sensitivity analysis plots, metaregression plots, and funnel plots for all results.

10.2196/80476 Multimedia Appendix 4 GRADE evidence quality assessment.

10.2196/80476 Checklist 1 PRISMA 2020 checklist.

## Abbreviations:

CBT: cognitive behavioral therapy
CNKI: China National Knowledge Infrastructure
DD: diabetes-related distress
DDS: Diabetes Distress Scale
DM: diabetes mellitus
GRADE: Grading of Recommendations Assessment, Development, and Evaluation
HbA_1c_: hemoglobin A_1c_
HKSJ: Hartung-Knapp-Sidik-Jonkman
ITT: intention-to-treat
PAID: Problem Areas in Diabetes
PI: prediction interval
PRISMA: Preferred Reporting Items for Systematic Reviews and Meta-Analyses
RCT: randomized controlled trial
SMD: standardized mean difference
T1DM: type 1 diabetes mellitus
T2DM: type 2 diabetes mellitus
Tele-CBT: tele–cognitive behavioral therapy
