# Supplementary material for: Tele–Cognitive Behavioral Therapy for the Treatment of Diabetes-Related Distress in Individuals With Diabetes Mellitus: Systematic Review and Meta-Analysis of Randomized Controlled Trials
Source: J Med Internet Res. 2025 Dec 24;27:e80476. doi: 10.2196/80476 (PMC12736637; doi:10.2196/80476)
Supplement: Multimedia Appendix 1 [file jmir-v27-e80476-s001.docx]

**Multimedia Appendix 1.** Search strategy.

| Database | Search strategy | Results |
| --- | --- | --- |
| Pubmed (NCBI) | ((((Diabetes mellitus[Title/Abstract] OR Diabetes[Title/Abstract] OR Type 1 diabetes[Title/Abstract] OR Type 2 diabetes[Title/Abstract]) OR ("Diabetes Mellitus"[Mesh])) AND (("Cognitive Behavioral Therapy"[Mesh]) OR (Cognitive behavio* therap*[Title/Abstract] OR Therapy, cognitive behavio*[Title/Abstract] OR Behavio* therapies, cognitive[Title/Abstract] OR Cognitive behavio* therapies[Title/Abstract] OR Therapies, cognitive behavio*[Title/Abstract] OR CBT[Title/Abstract] OR CCBT[Title/Abstract] OR ECBT[Title/Abstract] OR cognit* behavio* therap* OR cognit* therap*[Title/Abstract] OR cognitive psychotherap*[Title/Abstract] OR behavio*therap*[Title/Abstract] OR Problem-solving therapy[Title/Abstract] OR Behavioral activation[Title/Abstract]))) AND ((((((("Internet"[Mesh]) OR "Therapy, Computer-Assisted"[Mesh]) OR "Mobile Applications"[Mesh]) OR "Telemedicine"[Mesh]) OR "Distance Counseling"[Mesh]) OR (internet[Title/Abstract] OR mobile applications[Title/Abstract] OR telemedicine[Title/Abstract] OR distance counseling[Title/Abstract] OR ehealth[Title/Abstract] OR online*[Title/Abstract] OR web[Title/Abstract] OR tablet*[Title/Abstract] OR compute*[Title/Abstract])))) AND (Randomized controlled trial[Publication Type] OR Randomized controlled trial[Title/Abstract]) | 239 |
| Embase | ('Diabetes Mellitus'/exp OR 'Diabetes mellitus':ti,ab,kw OR 'Diabetes':ti,ab,kw OR 'Type 1 diabetes':ti,ab,kw OR 'Type 2 diabetes':ti,ab,kw) AND ('Cognitive Behavioral Therapy'/exp OR 'Cognitive behavio* therap*':ti,ab,kw OR 'Therapy, cognitive behavio*':ti,ab,kw OR 'Behavio* therapies, cognitive':ti,ab,kw OR 'Cognitive behavio* therapies':ti,ab,kw OR 'Therapies, cognitive behavio*':ti,ab,kw OR 'CBT':ti,ab,kw OR 'ICBT':ti,ab,kw OR 'CCBT':ti,ab,kw OR 'ECBT':ti,ab,kw OR 'cognit* behavio* therap*':ti,ab,kw OR 'cognit* therap*':ti,ab,kw OR 'cognitive psychotherap*':ti,ab,kw OR 'behavio*therap*':ti,ab,kw OR 'Problem-solving therapy':ti,ab,kw OR 'Behavioral activation':ti,ab,kw) AND ('Internet'/exp OR 'Therapy, Computer-Assisted'/exp OR 'Mobile Applications'/exp OR 'Telemedicine'/exp OR 'Distance Counseling'/exp OR 'internet':ti,ab,kw OR 'mobile applications':ti,ab,kw OR 'telemedicine':ti,ab,kw OR 'distance counseling':ti,ab,kw OR 'ehealth':ti,ab,kw OR 'online*':ti,ab,kw OR 'web':ti,ab,kw OR 'tablet*':ti,ab,kw OR 'compute*’:ti,ab,kw) AND ('controlled study'/de OR 'randomized controlled trial'/de) | 339 |
| Web of Science | TI=(Diabetes mellitus OR Diabetes OR Type 1 diabetes OR Type 2 diabetes) AND TS=(Cognitive behavio* therap* OR Therapy, cognitive behavio* OR Behavio* therapies, cognitive OR Cognitive behavio* therapies OR Therapies, cognitive behavio* OR CBT OR ICBT OR CCBT OR ECBT OR cognit* behavio* therap* OR cognit* therap* OR cognitive psychotherap* OR behavio*therap* OR Problem-solving therapy OR Behavioral activation) AND TS=(internet OR mobile applications OR telemedicine OR distance counseling OR ehealth OR online* OR web OR tablet* OR compute*) AND TS=(Randomized controlled trial ) | 249 |
| Scopus | TITLE-ABS-KEY ( "Diabetes mellitus" OR "Diabetes" OR "Type 1 diabetes" OR "Type 2 diabetes" ) AND TITLE-ABS-KEY ( "Cognitive behavio* therap*" OR "Therapy, cognitive behavio*" OR "Behavio* therapies, cognitive" OR "Cognitive behavio* therapies" OR "Therapies, cognitive behavio*" OR "CBT" OR "ICBT" OR "CCBT" OR "ECBT" OR "cognit* behavio* therap*" OR "cognit* therap*" OR "cognitive psychotherap*" OR "behavio*therap*" OR "Problem-solving therapy" OR "Behavioral activation" ) AND TITLE-ABS-KEY ( "internet" OR "mobile applications" OR "telemedicine" OR "distance counseling" OR "ehealth" OR "online*" OR "web" OR "tablet*" OR "compute*" ) AND ALL ( "Randomized controlled trial" ) | 307 |
| Cochrane Library | #1: MeSH descriptor: [Diabetes Mellitus] explode all trees  #2: (Diabetes mellitus OR Diabetes OR Type 1 diabetes OR Type 2 diabetes):ti,ab,kw (Word variations have been searched)  #3: #1 OR #2  #4: (internet OR mobile applications OR telemedicine OR distance counseling OR ehealth OR online* OR web OR tablet* OR compute*):ti,ab,kw (Word variations have been searched)  #5: (Cognitive behavio* therap* OR Therapy, cognitive behavio* OR Behavio* therapies, cognitive OR Cognitive behavio* therapies OR Therapies, cognitive behavio* OR CBT OR ICBT OR CCBT OR ECBT OR cognit* behavio* therap* OR cognit* therap* OR cognitive psychotherap* OR behavio*therap* OR Problem-solving therapy OR Behavioral activation):ti,ab,kw  #6: (Randomized controlled trial OR randomized OR placebo):ti,ab,kw  #7: #3 AND #4 AND #5 AND #6 | 386 |
| PsycINFO (Ovid) | TX (Diabetes mellitus OR Diabetes OR Type 1 diabetes OR Type 2 diabetes) AND TX (cognitive behavio* therap* OR Therapy, cognitive behavio* OR Behavio* therapies, cognitive OR Cognitive behavio* therapies OR Therapies, cognitive behavio* OR CBT OR ICBT OR CCBT OR ECBT OR cognit* behavio* therap* OR cognit* therap* OR cognitive psychotherap* OR behavio*therap* OR Problem-solving therapy OR Behavioral activation) AND TX (internet OR mobile applications OR telemedicine OR distance counseling OR ehealth OR online* OR web OR tablet* OR compute*) AND TX (randomized controlled trials or rtc or randomised control trials or clinical controlled trial or clinical trial) | 53 |
| CNKI | SU=( 认知行为疗法 + 认知行为疗法干预 + 认知行为疗法理论 + CBT + 远程认知行为疗法 + 网络认知行为疗法 + 问题解决疗法 + 认知治疗 + 认知心理疗法) AND SU=( 糖尿病 + DM + 1型糖尿病 + 2型糖尿病 ) AND SU=( 移动健康 + 移动应用 + APP + 网络 + 互联网 + 电子干预 + 电话 + 短信 + 电子邮件 + 远程 + 电脑 + 数字技术 ) | 21 |
| VIP | M=(认知行为疗法 OR 认知行为疗法干预 OR 认知行为疗法理论 OR CBT OR 远程认知行为疗法 OR 网络认知行为疗法 OR 问题解决疗法 OR 认知治疗 OR 认知心理疗法) AND M=(糖尿病 OR DM OR 1型糖尿病 OR 2型糖尿病) AND R=(移动健康 OR 移动应用 OR APP OR 网络 OR 互联网 OR 电子干预 OR 电话 OR 短信 OR 电子邮件 OR 远程 OR 电脑 OR 数字技术) | 11 |
| CBM | (( "认知行为疗法"[不加权:扩展]) OR ( "认知行为疗法"[常用字段:智能] OR "认知行为疗法干预"[常用字段:智能] OR "认知行为疗法理论"[常用字段:智能] OR "CBT"[常用字段:智能] OR "远程认知行为疗法"[常用字段:智能] OR "网络认知行为疗法"[常用字段:智能] OR "问题解决疗法"[常用字段:智能] OR "认知治疗"[常用字段:智能] OR "认知心理疗法"[常用字段:智能] )) AND (("糖尿病"[不加权:扩展] OR "糖尿病, 2型"[不加权:扩展] OR "糖尿病, 2型"[不加权:扩展] OR "糖尿病, 2型"[不加权:扩展] OR "糖尿病, 1型"[不加权:扩展] OR "糖尿病, 1型"[不加权:扩展] OR "糖尿病, 1型"[不加权:扩展]) OR ( "糖尿病"[常用字段:智能] OR "DM"[常用字段:智能] OR "1型糖尿病"[常用字段:智能] OR "2型糖尿病"[常用字段:智能])) AND (( "互联网使用"[不加权:扩展] OR "社会化媒体"[不加权:扩展] OR "社交网络"[不加权:扩展] OR "远程医学"[不加权:扩展] OR "电话"[不加权:扩展] OR "移动应用"[不加权:扩展] ) OR ( "移动健康"[常用字段:智能] OR "移动应用"[常用字段:智能] OR "APP"[常用字段:智能] OR "网络"[常用字段:智能] OR "互联网"[常用字段:智能] OR "电子干预"[常用字段:智能] OR "电话"[常用字段:智能] OR "短信"[常用字段:智能] OR "电子邮件"[常用字段:智能] OR "远程"[常用字段:智能] OR "电脑"[常用字段:智能] OR "数字技术"[常用字段:智能])) | 15 |
| WANGFANG | KW=(认知行为疗法 OR 认知行为疗法干预 OR 认知行为疗法理论 OR CBT OR 远程认知行为疗法 OR 网络认知行为疗法 OR 问题解决疗法 OR 认知治疗 OR 认知心理疗法) AND TI=(糖尿病 OR DM OR 1型糖尿病 OR 2型糖尿病) AND AB=(移动健康 OR 移动应用 OR APP OR 网络 OR 互联网 OR 电子干预 OR 电话 OR 短信 OR 电子邮件 OR 远程 OR 电脑 OR 数字技术) | 43 |

Note: The last search dates for the above databases were all September 25, 2025.
