# Supplementary material for: Tele–Cognitive Behavioral Therapy for the Treatment of Diabetes-Related Distress in Individuals With Diabetes Mellitus: Systematic Review and Meta-Analysis of Randomized Controlled Trials
Source: J Med Internet Res. 2025 Dec 24;27:e80476. doi: 10.2196/80476 (PMC12736637; doi:10.2196/80476)
Supplement: Multimedia Appendix 2 [file jmir-v27-e80476-s002.docx]

**Multimedia Appendix 2**

**Table S1.** Basic characteristics of included literature.

| **Authors (Year)** | **Participant Demographics[Age (I/C) ], Source of participants, sample Size (I/C), diabetes-related characteristics, depressive symptoms, and comorbidity** | **Intervention details** | | **Control** | **outcome indicator** | **ITT** |
| --- | --- | --- | --- | --- | --- | --- |
|  |  | **Intervention content, Form of intervention, Therapist** | **Intervention frequency, duration and Length of follow-up** |  |  |  |
| Bae et al (2024) [47] | Country: Korea  Age: 60.4±4.3; 63.9±5.1  Sex: female (51.3%), male (48.7%)  Source of participants: public health centers  Sample size: 39(20/19)  Types of diabetes: T2DM  Diabetes duration (years): 6.5; 13.0  Depressive symptoms: mild-to-moderate depressive  Comorbidities: diabetes complications (28.2%) | Content: application developed based on CBT's core content behavioral activation, consisting of seven modules  Form: mobile application  Therapist: psychologist | Frequency: N/A  Duration: 12 weeks  Follow-up: 16 weeks | Usual care | DD: DDS  Depressive: PHQ-9  Anxieties: GAD-7  HbA1c | Yes |
| Carreira et al (2023) [48] | Country: Spain  Age: 37.48±11.01; 35.03±12.87  Sex: female (69.2%), male (30.8%)  Source of participants: hospitals and health service centers  Sample size: 65(35/30)  Types of diabetes: T1DM  Time with diabetes (years): 18.71±12.72; 21.26±12.82  Depressive symptoms: mild-moderate depressive  Comorbidities: diabetes or other complications (58.5%) | Content: internet-based CBT program with 9 online modules  Form: internet-based programs  Therapist: psychologist | Frequency: once a week  Duration: 9 weeks  Follow-up: N/A | Usual care | DD: DDS  Depressive: BDI  Anxieties: STAI  HbA1c | N/A |
| Clarke et al (2019) [20] | Country: Australia  Age: 57.7±10.6; 57.7±10.0  Sex: female (64.3%), male (35.7%)  Source of participants: public health centers  Sample size: 723(368/355)  Types of diabetes: T2DM  Age at diagnosis diabetes (years): 46.6±11.1; 47.2±10.9  Depressive symptoms: positive for depression  Comorbidities:: N/A | Content: fully automated, self-directed CBT-based web-based program-guided e-mental health  Form: web-based program  Therapist: not involved | Frequency: N/A  Duration: 3 months  Follow-up: 3 months | Placebo group | DD: DDS  Depressive: PHQ-9  Anxieties: GAD-7 | Yes |
| Groeneveld et al (2024) [49] | Country: Netherlands  Age: 67.7 ± 8.4; 71.8 ± 7.3  Sex: female (33.3%), male (66.7%)  Source of participants: Hoorn Diabetes Care System  Sample size: 57(29/28)  Types of diabetes: T2DM  Diabetes duration (years): 13.4±4.5; 15.2±6.0  Depressive symptoms: 33.3% have symptoms of depression  Comorbidities:: N/A | Content: web-based 5-week structured CBT program with 5 online modules  Form: online  Therapist: trained doctoral students | Frequency: each lessons is one hour long  Duration: 8 weeks  Follow-up: 6 months | Usual care | depressive: PHQ-9  HbA1c | Yes |
| Holloway et al (2025) [50] | Country: Australia  age: 53; 54  Sex: female (58.9%), male (40.2%), Nonbinary (0.7%), Not stated (0.2%)  Source of participants: the National Diabetes Services Scheme  Sample size: 428(215/213)  Types of diabetes: T1DM or T2DM  Diabetes duration (years): 9; 11  Depressive symptoms: only includes mild depression  Comorbidities: Heart disease/myocardial infarction (11.7%); Neuropathy (11.7%);  Kidney damage/renal failure (9.8%); Retinopathy (12.6%); Vascular disease (7.7%); Sexual dysfunction (10.5%) | Content: low-intensity CBT intervention with PST psychoeducation, up to 4 telemedicine  Form: telehealth Enabled Network  Therapist: professional guidance from non-traditional psychotherapists | Frequency: sessions of 45-60 minutes each, spaced one week apart  Duration: 12 weeks  Follow-up: N/A | Usual care | DD: PAID | Yes |
| Newby et al (2017) [29] | Country: Australia  Age: 43.5±13.3; 49.3±11.5  Sex: female (71%), male (29%)  Source of participants: public health centers  Sample size: 90(41/49)  Types of diabetes: T1DM or T2DM  Age of onset (diabetes): 27.6±18.1; 32.9±16.2  Depressive symptoms: only includes mild-moderate depression  Comorbidities: Asthma (31%);  Cancer (7%); Stroke (3%);  Circulatory condition (48%) Gout or rheumatism or arthritis (37%) | Content: internet-based CBT consisting of 6 automated online modules  Form: web-based with no face-to-face components  Therapist: clinical Psychologist and Registered Psychiatrist | Frequency: 1 per week for 10 weeks  Duration: 10 weeks  Follow-up: 3 months | Usual care | DD: PAID  Depressive: PHQ-9  Anxieties: GAD-7  HbA1c | Yes |
| Nobis et al (2015) [51] | Country: Germany  Age: 50±12; 51±12  Sex: female (63.0%), male (37.0%)  Source of participants: public health centers  Sample size: 256(129/127)  Types of diabetes: T1DM or T2DM  Diabetes duration: 3-12 months (7%); 1-10 years (46.5%); >10 years (46.5%)  Depressive symptoms: moderate and high depressive symptoms  Comorbidities: diabetes complications: (25%) | Content: web-based intervention and cell phone support for CBT core behavioral intervention consisting of six consecutive sessions, with the opportunity of two additional sessions  Form: web-based intervention  Therapist: standardized instruction for coaches | Frequency: one session per week; each session lasted ~45 min.  Duration: 8 weeks  Follow-up: N/A | Usual care | DD: PAID  Depressive: CES-D | Yes |
| Piette et al (2011) [52] | Country: US  age: 56.0±10.9; 55.1±9.4  Sex: female (51.5%), male (48.5%)  Source of participants: healthcare system  Sample size: 291(145/146)  Types of diabetes: T2DM  Diabetes duration: NA  Depressive symptoms: significant depressive symptoms (BDI scores ≥14)  Comorbidities: N/A | Content: the telephone-based CBT program  Form: telephonic counseling  Therapist: instruction provided by nurses trained in standardized CBT | Frequency: an initial intensive phase of 12-weekly sessions followed by nine monthly booster sessions  Duration: 12 months  Follow-up: N/A | Usual care | Depressive: BDI | Yes |
| Schlicker et al (2019) [53] | Country: Germany  Age: 50.16±11.68; 51.34±11.92  Sex: female (62.8%), male (37.2%)  Source of participants: public health centers  Sample size: 253(127/126)  Types of diabetes: T1DM or T2DM  Average years since diabetes diagnosis: 5-10 years  Depressive symptoms: moderate or high depressive symptoms  Comorbidities: secondary diseases (24.9%) | Content: internet-based CBT for depression and diabetes.six minimally guided sessions with approximately one session à 45 to 60 minutes per week. There are two optional sessions and one booster session, after completion of the intervention.  Form: internet-based  Therapist: psychologists provide feedback via email | Frequency:once a week for six weeks  Duration: 6 weeks  Follow-up: 6 months | Online psychology | DD: PAID  Depressive: BDI  HbA1c | Yes |
| Stadler et al (2025) [54] | Country: UK  Age: 35.5; 34.4  Sex: female (97.5%), male (2.5%)  Source of participants: King’s College Hospital  Sample size: 40 (20/20)  Types of diabetes: T1DM  Diabetes duration (years): 16.0; 24.5  Depressive symptoms: exclude severe depression  Comorbidities: N/A | Content: based CBT, including 12 one-on-one virtual sessions  Form: videoconference and mobile app  Therapist: guidance for CBT-trained diabetes specialist nurses | Frequency: the first four therapy sessions were conducted  weekly to fortnightly, and the final eight sessions were scheduled at times which suited the participant’s needs (weekly to monthly intervals)  Duration: 6 months  Follow-up: 12 months | Usual care | DD: DDS  Depressive: PHQ-9  Anxieties: GAD-7 | Yes |
| Vaughan et al (2022) [55] | Country: US  age: 61.9±8.3  Sex: female (10.2%), male (89.8%)  Source of participants: hospital-based primary care clinics and community-based outpatient clinics  Sample size: 225(136/89)  Types of diabetes: T1DM or T2DM uncontrolled diabetes  Diabetes duration (years): 16.0; 24.5  Depressive symptoms: significant depressive symptoms (PHQ-9 ≥ 10）  Comorbidities: N/A | Content: based CBT-oriented behavioral health intervention.  received a collaborative, goal-setting ‘‘active’’ intervention for the first 6 months. Then they received three bimonthly maintenance sessions  Form: telephone-based telehealth intervention  Therapist: health coach | Frequency: The first 6 months that occurred biweekly (30–40min) for sessions 1–3 and monthly (15min) for sessions 4–6. Months 7-12 are the maintenance phase, with three bimonthly maintenance sessions of 15 minutes each.  Duration: 6 months  Follow-up: 6 months | Enhancing usual care | DD: PAID | Yes |

Note: US: United States; UK: United Kingdom; N/A: not available;① DDS: Diabetes Distress Scale; ② PAID: Problem Areas in Diabetes Scale; ③ PHQ-9: 9-item Patient Health Questionnaire Scale; ④ BDI: Beck Depression Inventory;⑤ CES-D: Center for Epidemiologic Studies Depression Scale; ⑥ GAD-7: Generalized Anxiety Disorder Scale ⑦ STAI: State-Trait Anxiety Inventory
