# Supplementary material for: Tele–Cognitive Behavioral Therapy for the Treatment of Diabetes-Related Distress in Individuals With Diabetes Mellitus: Systematic Review and Meta-Analysis of Randomized Controlled Trials
Source: J Med Internet Res. 2025 Dec 24;27:e80476. doi: 10.2196/80476 (PMC12736637; doi:10.2196/80476)
Supplement: Multimedia Appendix 3 [file jmir-v27-e80476-s003.docx]

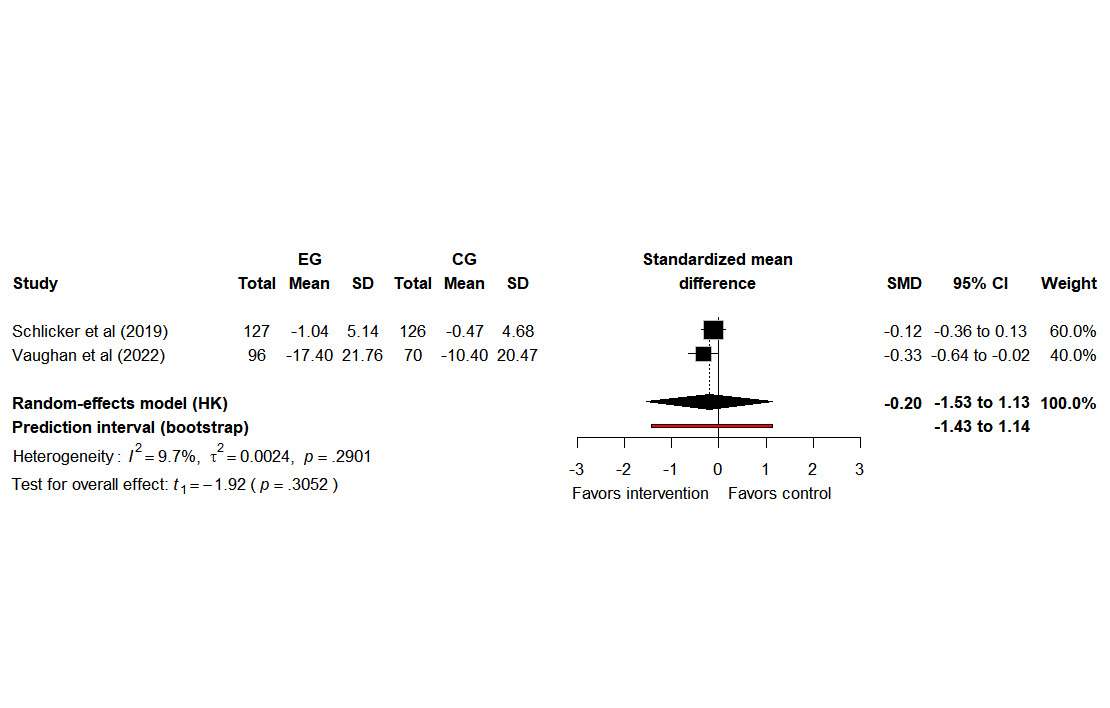


**Figure S1**. Forest plot of diabetes-related distress results in follow-up assessments.


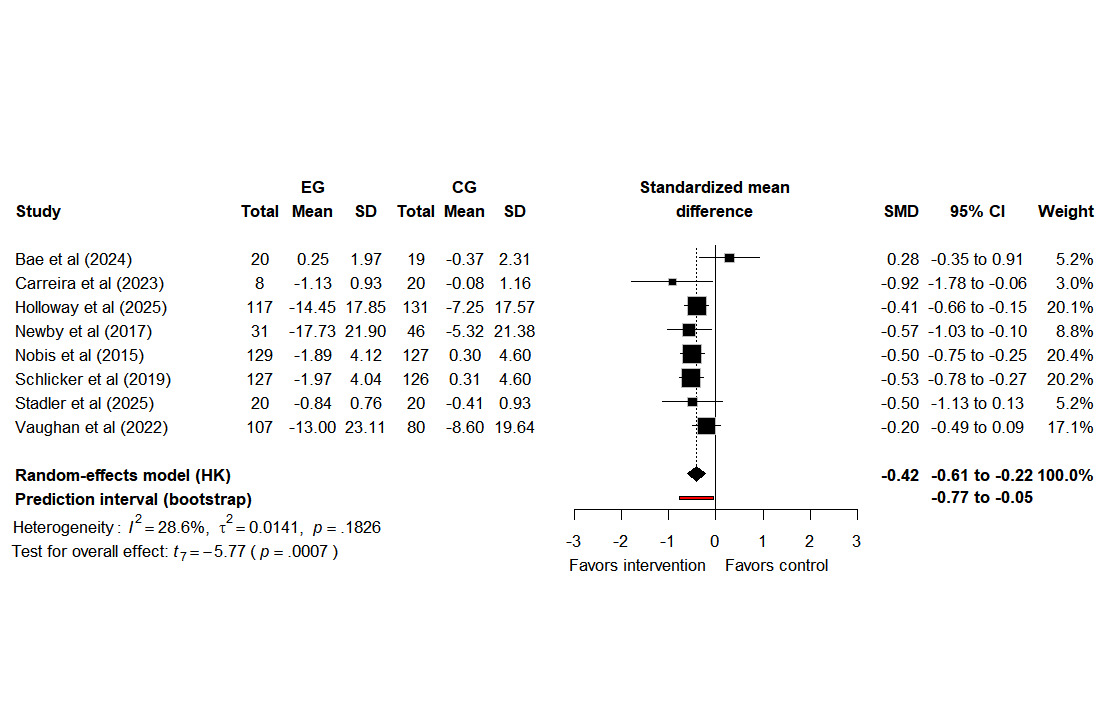


**Figure S2.** Forest plot of diabetes-related distress after excluding a single study.


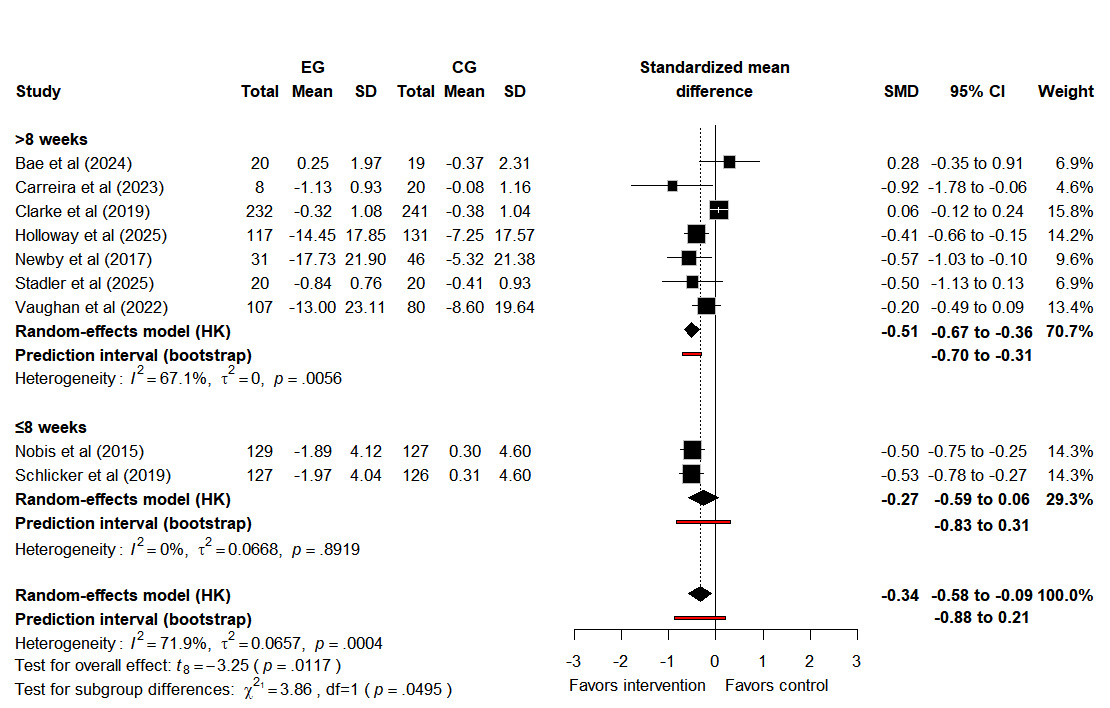


**Figure S3.** Forest plot of diabetes-related distress results in the intervention duration subgroup analysis.


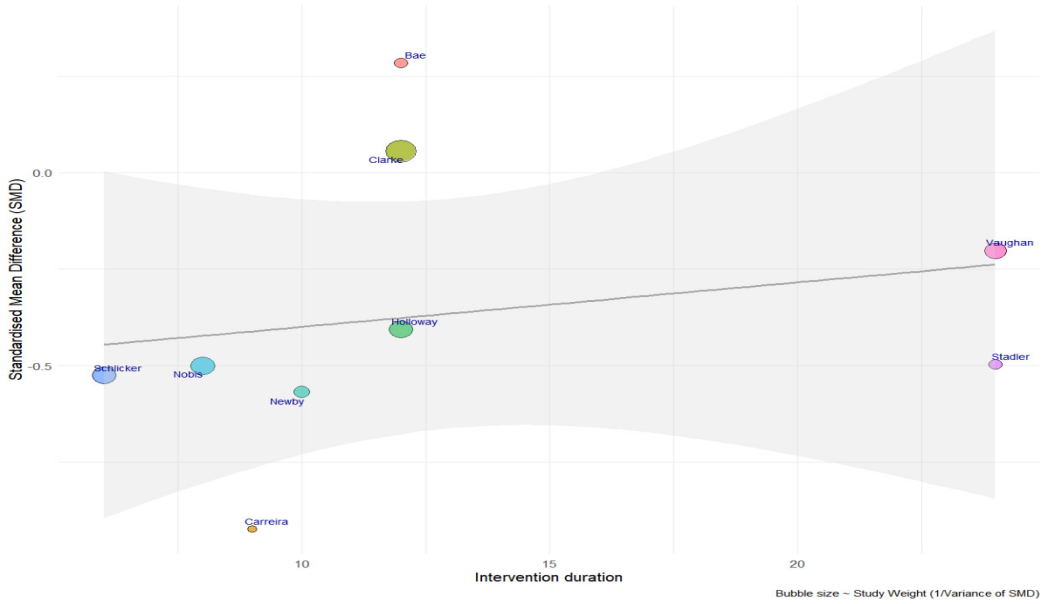


**Figure S4.** Bubble plot of meta-regression diabetes-related distress results by intervention duration.


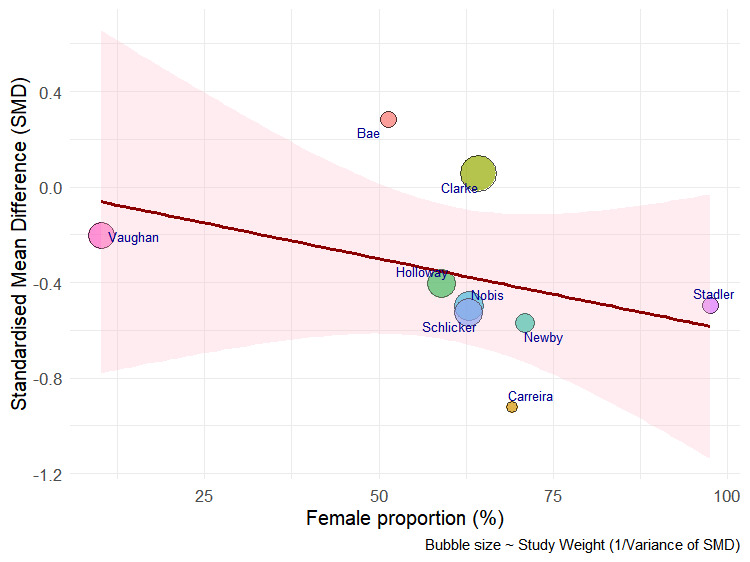


**Figure S5.** Bubble plot of meta-regression diabetes-related distress results by female proportion.


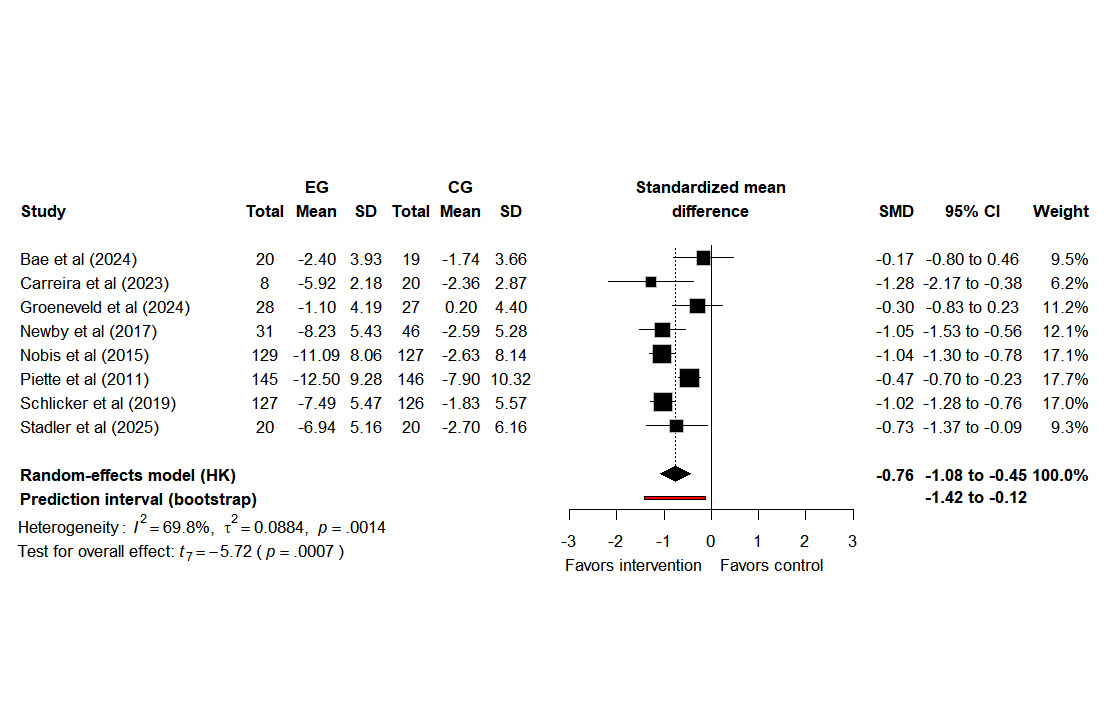
**Figure S6.** Forest plot of depression after excluding a single study.


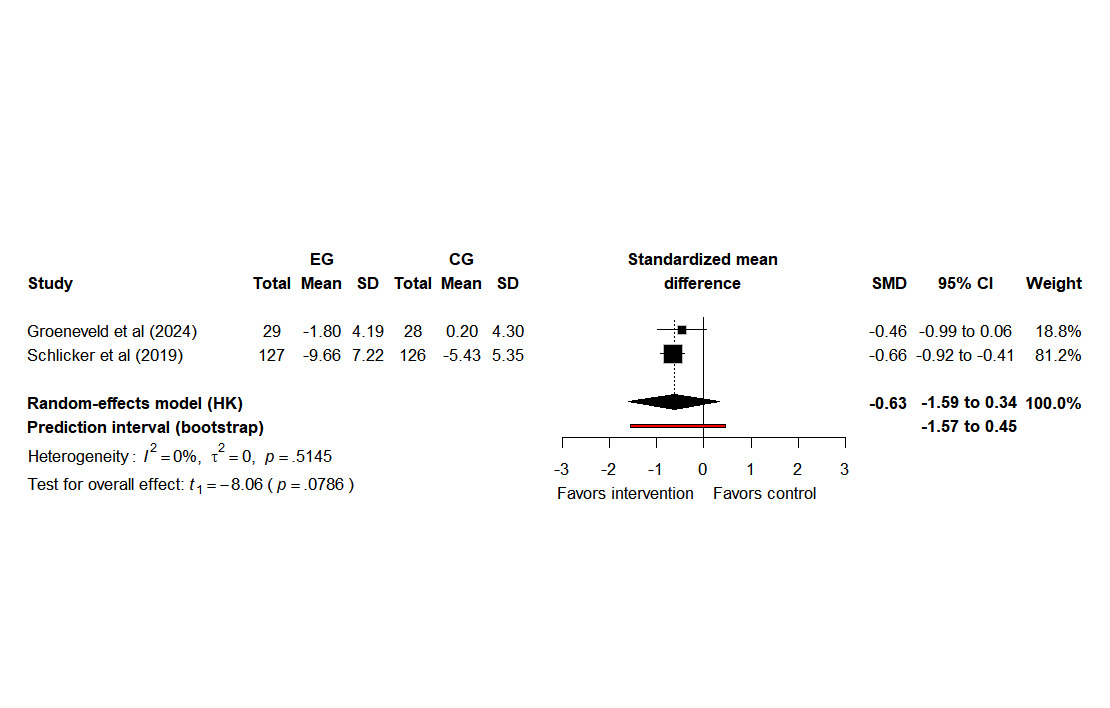


**Figure S7.** Forest plot of follow-up assessments of depression outcomes.

**
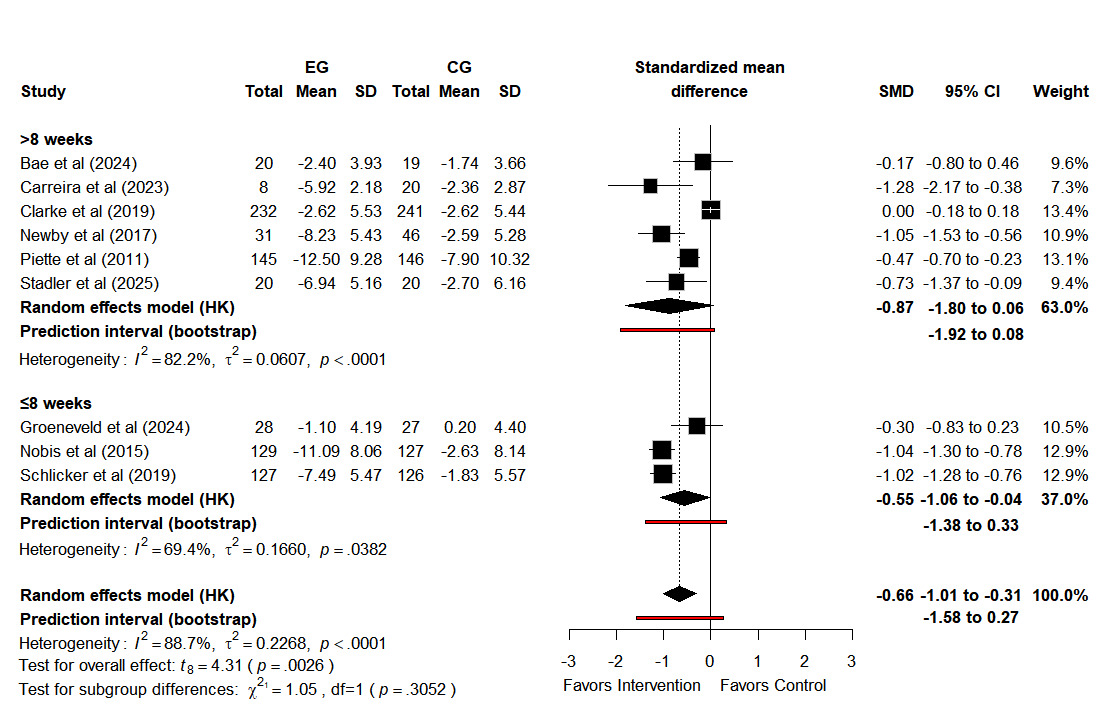
**

**Figure S8.** Forest plot of depression results in the intervention duration subgroup analysis.


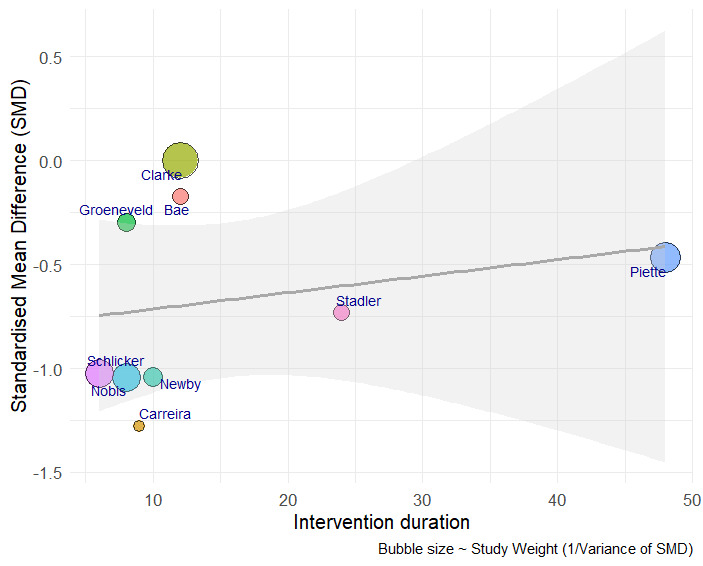


**Figure S9.** Bubble plot of meta-regression results on depression by intervention duration.


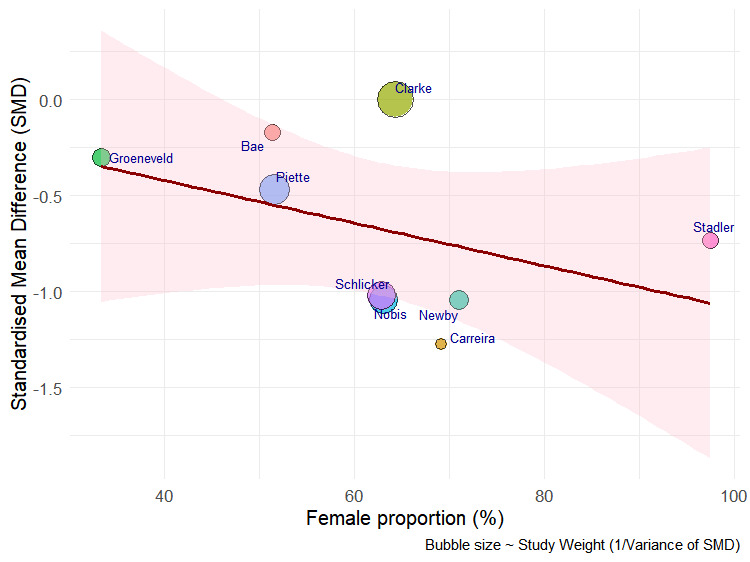


**Figure S10.** Bubble plot of meta-regression results on depression by female proportion.


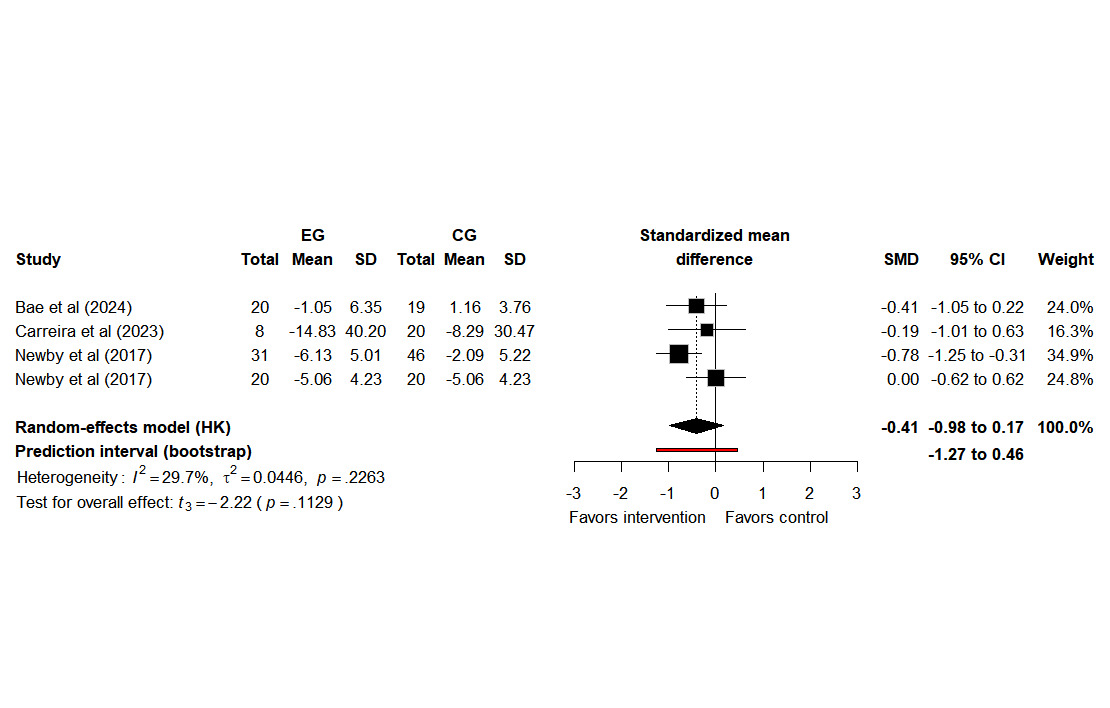


**Figure S11.** Forest plot of anxiety after excluding a single study.


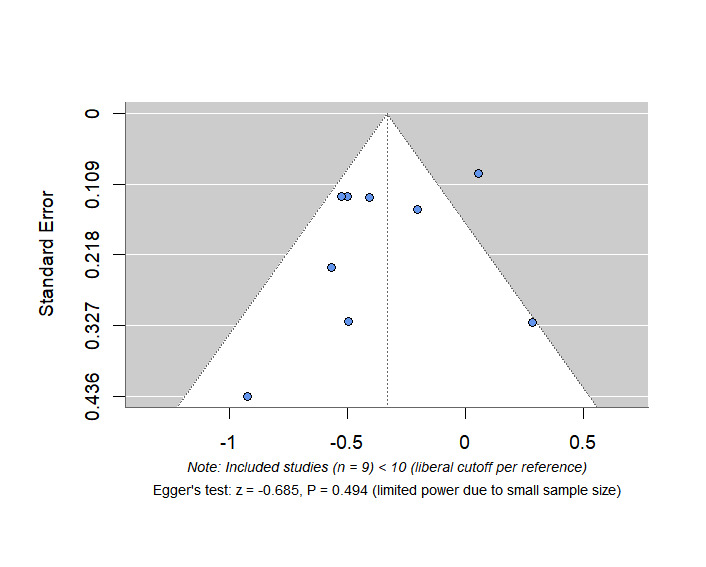


**Figure S12.** Funnel plot of diabetes-related distress outcomes.


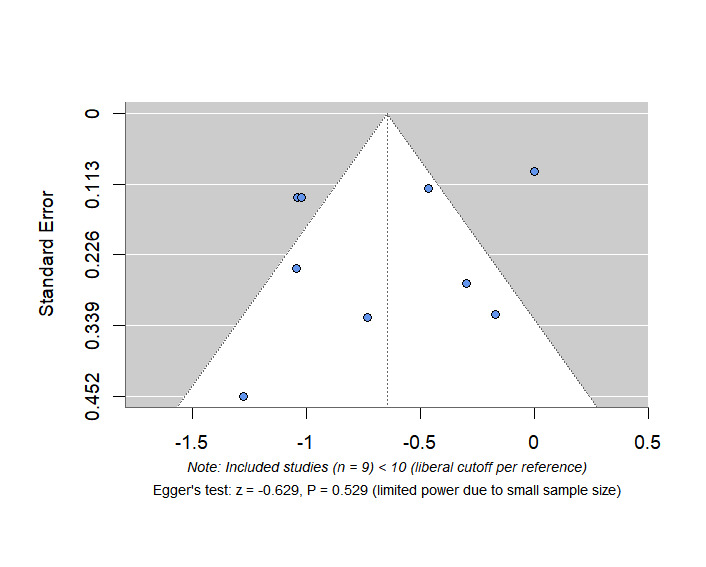


**Figure S13.** Funnel plot of depression outcomes.

**
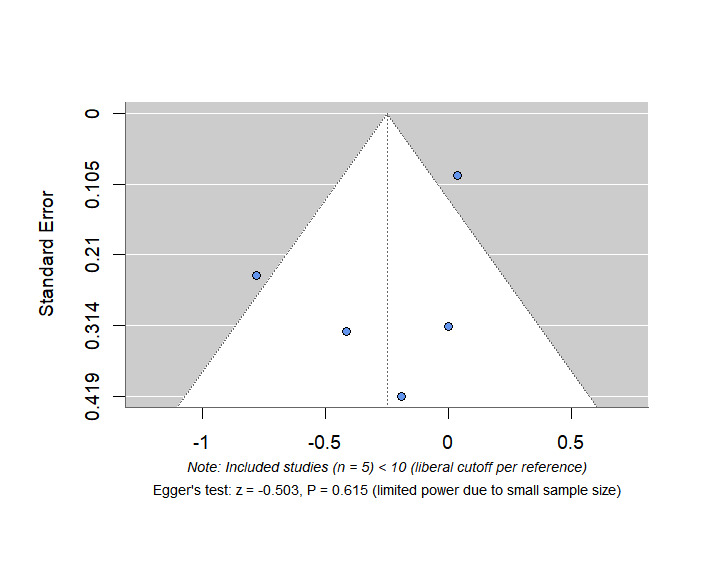
**

**Figure S14.** Funnel plot of anxiety outcomes.

**
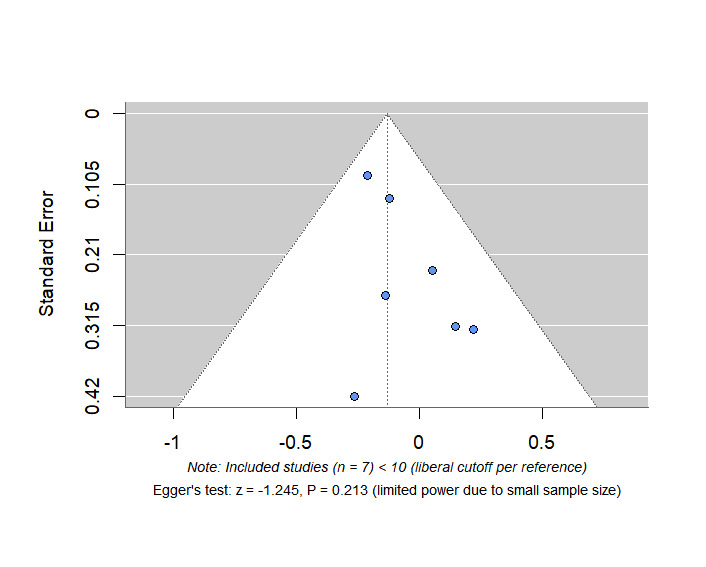
**

**Figure S15.** Funnel plot of HbA1c outcomes.
