# Supplementary material for: Tele–Cognitive Behavioral Therapy for the Treatment of Diabetes-Related Distress in Individuals With Diabetes Mellitus: Systematic Review and Meta-Analysis of Randomized Controlled Trials
Source: J Med Internet Res. 2025 Dec 24;27:e80476. doi: 10.2196/80476 (PMC12736637; doi:10.2196/80476)
Supplement: Multimedia Appendix 4 [file jmir-v27-e80476-s004.docx]

**Author(s):** Xiaohong Xu, Fang Wang, Shunqi Liao, Jingxian Liu, Lingyi Xiao.

**Question:** Tele-cognitive behavioral therapy for the treatment of diabetes-Related distress in individuals with diabetes mellitus

**Setting:** Hospitals, nursing homes, communities, and homes

**Bibliography:**

| **Certainty assessment** | | | | | | | **№ of patients** | | **Effect** | | **Certainty** | **Importance** |
| --- | --- | --- | --- | --- | --- | --- | --- | --- | --- | --- | --- | --- |
| **№ of studies** | **Study design** | **Risk of bias** | **Inconsistency** | **Indirectness** | **Imprecision** | **Other considerations** | **Tele-Cognitive Behavioral Therapy** | **[comparison]** | **Relative (95% CI)** | **Absolute (95% CI)** |  |  |
| **Diabetes-related distress (assessed with: Problem Areas in Diabetes scale or Diabetes Distress Scale)** | | | | | | | | | | | | |
| 9 | randomised trials | serious^a,b,c^ | serious^d^ | not serious | serious^e^ | none | 791 | 810 | - | SMD -**0.33 SD lower** (-0.57 lower to -0.1 lower) | ⨁◯◯◯ Very low^a,b,c,d,e^ | CRITICAL |
| **Depression symptoms** | | | | | | | | | | | | |
| 9 | randomised trials | serious^a,b,c^ | not serious | not serious | serious^e^ | none | 740 | 742 | - | SMD -**0.65 SD lower** (-0.99 lower to -0.3 lower) | ⨁⨁◯◯ Low^a,b,c,e^ | CRITICAL |
| **Hemoglobin A1c** | | | | | | | | | | | | |
| 7 | randomised trials | serious^a,b,c^ | serious^f^ | not serious | serious^e^ | none | 466 | 499 | - | SMD -**0.13 SD lower** (-0.25 lower to -0.01 lower) | ⨁◯◯◯ Very low^a,b,c,e,f^ | CRITICAL |

**CI:** confidence interval; **SMD:** standardised mean difference

#### Explanations

a. Since remote CBT is a type of psychological intervention, it is difficult for researchers to blind participants and implementers; none of the studies conducted blinding, posing a high risk of performance bias.

b. Outcome indicators are mainly measured through subjective scales, and there is also uncertainty regarding the risk of bias in outcome measurement.

c. Most studies did not adequately describe allocation concealment and outcome assessor blinding.

d. Differences in research design, such as intervention measures, study populations, and outcome indicators, have led to one study showing significant inconsistencies in results compared to the others.

e. In terms of sample size, there are significant differences among the studies. Some studies have relatively small sample sizes, which may lead to wider confidence intervals for effect estimates, making the estimates less precise.

f. There are significant inconsistencies in the results between different studies.
